# Localized strain characterization of cardiomyopathy in Duchenne muscular dystrophy using novel 4D kinematic analysis of cine cardiovascular magnetic resonance

**DOI:** 10.1186/s12968-023-00922-3

**Published:** 2023-02-16

**Authors:** Conner C. Earl, Victoria I. Pyle, Sydney Q. Clark, Karthik Annamalai, Paula A. Torres, Alejandro Quintero, Frederick W. Damen, Kan N. Hor, Larry W. Markham, Jonathan H. Soslow, Craig J. Goergen

**Affiliations:** 1 Weldon School of Biomedical Engineering, Purdue University, 206 S. Martin Jischke Dr., West Lafayette, IN 47907 USA; 2 Division of Pediatric Cardiology, Riley Children’s Hospital at Indiana University Health, Indianapolis, IN USA; 3 Indiana University School of Medicine, Indianapolis, IN USA; 4 The Heart Center, Nationwide Children’s Hospital, Ohio State University, Columbus, OH USA; 5 Division of Pediatric Cardiology, Department of Pediatrics, Vanderbilt University Medical Center, Nashville, TN USA

**Keywords:** Duchenne muscular dystrophy, Cardiomyopathy, Strain, Cardiac magnetic resonance, 3D, 4D, Cardiac biomechanics

## Abstract

**Background:**

Cardiomyopathy (CMP) is the most common cause of mortality in Duchenne muscular dystrophy (DMD), though the age of onset and clinical progression vary. We applied a novel 4D (3D + time) strain analysis method using cine cardiovascular magnetic resonance (CMR) imaging data to determine if localized strain metrics derived from 4D image analysis would be sensitive and specific for characterizing DMD CMP.

**Methods:**

We analyzed short-axis cine CMR image stacks from 43 DMD patients (median age: 12.23 yrs [10.6–16.5]; [interquartile range]) and 25 male healthy controls (median age: 16.2 yrs [13.3–20.7]). A subset of 25 male DMD patients age-matched to the controls (median age: 15.7 yrs [14.0-17.8]) was used for comparative metrics. CMR images were compiled into 4D sequences for feature-tracking strain analysis using custom-built software. Unpaired t-test and receiver operator characteristic area under the curve (AUC) analysis were used to determine statistical significance. Spearman’s rho was used to determine correlation.

**Results:**

DMD patients had a range of CMP severity: 15 (35% of total) had left ventricular ejection fraction (LVEF) > 55% with no findings of myocardial late gadolinium enhancement (LGE), 15 (35%) had findings of LGE with LVEF > 55% and 13 (30%) had LGE with LVEF < 55%. The magnitude of the peak basal circumferential strain, basal radial strain, and basal surface area strain were all significantly decreased in DMD patients relative to healthy controls (*p* < 0.001) with AUC values of 0.80, 0.89, and 0.84 respectively for peak strain and 0.96, 0.91, and 0.98 respectively for systolic strain rate. Peak basal radial strain, basal radial systolic strain rate, and basal circumferential systolic strain rate magnitude values were also significantly decreased in mild CMP (No LGE, LVEF > 55%) compared to a healthy control group (*p *< 0.001 for all). Surface area strain significantly correlated with LVEF and extracellular volume (ECV) respectively in the basal (rho = − 0.45, 0.40), mid (rho = − 0.46, 0.46), and apical (rho = − 0.42, 0.47) regions.

**Conclusion:**

Strain analysis of 3D cine CMR images in DMD CMP patients generates localized kinematic parameters that strongly differentiate disease from control and correlate with LVEF and ECV.

**Supplementary Information:**

The online version contains supplementary material available at 10.1186/s12968-023-00922-3.

## Background

Duchenne muscular dystrophy (DMD) is an X-linked recessive progressive neuromuscular disease with an incidence of approximately 1 in 5000 live male births [1–3]. DMD is caused by a mutation in the *DMD* gene resulting in severely reduced or absent functional dystrophin [4, 5]. A lack of dystrophin leads to a loss of sarcolemma integrity, triggering muscle degradation followed by necrosis, fibrosis, and fibro-fatty replacement of normal cardiac muscle tissue [6–9]. Dystrophin deficiency in the heart leads to myocardial necrosis and fibro-fatty replacement commonly resulting in a lethal cardiomyopathy (CMP), though the onset and progression of this phenotype vary [10–13]. In the current era, CMP is the most common cause of mortality in DMD, but imaging biomarkers are limited in their ability to predict the early onset or rate of CMP progression [14, 15]. Clinically important CMP typically becomes apparent in the middle of the second decade, affecting one-third of patients by age 14 and nearly all patients over 18 years of age [16, 17]. However, pre-clinical cardiac involvement is thought to be present in up to one-fourth of DMD patients under 6 years old [17].

Early identification of fibro-fatty replacement and myocardial damage allows for prophylactic treatment with cardioprotective medications for DMD CMP [1]. While several non-randomized studies have shown that glucocorticoids, angiotensin-converting enzyme inhibitors, and aldosterone inhibitors may delay CMP progression, [18–21] more recent trials suggest that these standard heart failure medications may not be as effective in DMD as in other forms of CMP [22]. Novel therapies are needed but cannot be developed without a better understanding of CMP progression and the identification of novel CMP biomarkers.

Over the past few years, we have developed methods for high-sensitivity spatiotemporal mapping of 4D (3D + time) gated cardiac echocardiographic data [23–26]. These advances have allowed us to identify subtle imaging biomarkers in a variety of cardiac disease animal models of myocardial infarction [27, 28], aortic aneurysm [29, 30], and atherosclerosis [31]. Applying these techniques to 3D + time cardiovascular magnetic resonance (CMR) images offers a promising method for biomechanical characterization of pathologic changes in DMD patients. The objective of this manuscript was to adapt our novel method of spatiotemporal mapping of 4D kinematic data to CMR cine images and to apply this new method to patients with DMD CMP. We hypothesized that this novel method of strain analysis would show significant differences between DMD CMP and healthy controls and that regional strain and strain rate would allow for stronger differentiation compared to global values alone.

## Methods

### Patient sampling

DMD CMP patients and healthy controls were selected from a prospective observational study approved by the Vanderbilt Institutional Review Board, all of which signed approved consents or assents. DMD CMP patients included in the original study had phenotypically diagnosed DMD confirmed through genetic testing or muscle biopsy with at least one CMR scan. Exclusion criteria included patients with a genetic diagnosis other than DMD and patients without late gadolinium enhancement (LGE) assessment or non-diagnostic LGE study. For our study, we also excluded two patients with poor image quality resulting in poor quality 3D reconstruction for strain analysis.

### Image acquisition

CMR images were obtained using a 1.5 T system (Avanto, Siemens Healthineers, Erlangen, Germany). Cine images in a short-axis stack and in the 4-chamber, 3-chamber, and 2-chamber views were acquired using balanced steady-state free-precession (bSSFP) imaging [32]. Typical parameters for imaging were 6–8 mm slice thickness, 340 mm × 340 mm field of view with a 256 × 192 matrix size, and minimum echo and repetition time. The resulting images included 11–17 short-axis cine slices for each patient with 6–8 mm thickness and 20–25 images spanning the cardiac cycle. A peripheral intravenous line was used to administer intravenous Gd-DTPA contrast (0.2 mmol/kg gadopentate dimeglumine, Magnevist®, Bayer Healthcare, Berlin, Germany or 0.15 mmol/kg gadobutrol, Gadovist®, Bayer Healthcare). We performed LGE imaging using single-shot inversion recovery bSSFP imaging with an optimized inversion time to null myocardium and phase-sensitive inversion recovery (PSIR) bSSFP imaging with an inversion time of 300 ms. A segmented inversion recovery turboFLASH sequence with optimized inversion recovery to null myocardial signal was also used.

Breath-held modified Look-Locker inversion recovery (MOLLI) sequences were performed as described [33–35] with typical imaging parameters. MOLLI sequences were motion corrected and a T1 map was generated on the scanner [36, 37]. A goodness of fit map was also performed at the time of the scan to evaluate data quality. Any image felt to be inadequate due to poor breath holds or poor motion correction was repeated at the time of the scan. T2 mapping using a breath-held, electrocardiogram (ECG)-triggered, bSSFP sequence with motion correction was performed in the short-axis prior to contrast administration in the same slice locations as the MOLLI sequences.

### CMR post-processing

All CMR post-processing was performed blinded to clinical data by an image analyst with all analyses verified by a cardiologist with 10 years of experience (JHS). Ventricular volumes and function were calculated using Medis QMass (MedisSuite 2.1, Medis, Leiden, The Netherlands). The presence or absence of LGE, as well as location using the standard 17-segment model [38] was qualitatively assessed. LGE severity was assessed as previously described [39].

T1 maps, obtained before and after contrast administration, as described by Messroghli et al. [33], were used along with the subject’s hematocrit to calculate an extracellular volume (ECV) map using manual registration in QMap from Medis. The ECV was calculated as described previously [32, 35] using pre- and post-contrast T1 along with patient hematocrit levels. Areas of LGE were included as these areas were felt to be the most focal areas in a continuum of diffuse ECM expansion [34].

### 3D + Time CMR image strain analysis

**Fig. 1:**
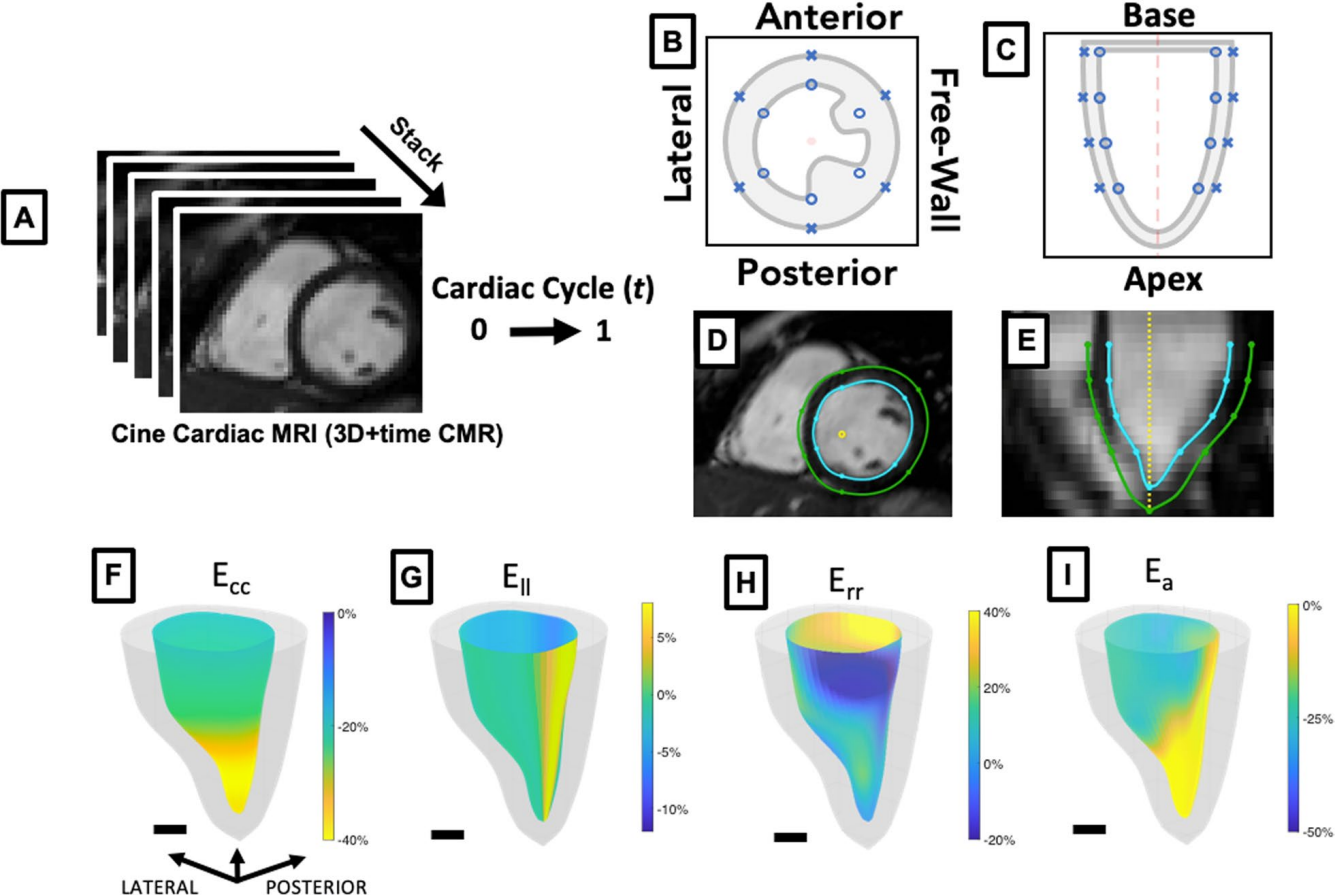
3D + time CMR localized strain analysis **A** 3D + time cardiovascular magnetic resonance (CMR) image created from 2D cine short -axis image stack. **B**, **C** 48 point left ventricular (LV) feature-tracking schematic with example images in short-axis (**D**) and long axis (**E**) views from a MATLAB-based graphical user interface. Example circumferential (E_cc_; **F**), longitudinal (E_ll_; **G**), radial (E_rr_; **H**), surface area (E_a_; **I**) colorized strain maps at peak systole derived from 3D + time CMR images of a Duchenne muscular dystrophy (DMD) patient from MATLAB feature-tracking analysis. **F**–**I** also correspond to Additional files 1, 2, 3, 4. Scale bar = 1 cm

To prepare image data for strain analysis, we compiled all short-axis cine CMR DICOM images into a 3D + time data set using MATLAB (R2020a, Mathworks, Natick, Massachusetts, USA). Using a custom-built graphical user interface, we then oriented the data along a centerline longitudinal axis. Following this step, we estimated the basal and apical motion throughout the cardiac cycle along the longitudinal axis as described previously [24, 40]. When base and apex tracking was inhibited by the low spatial resolution along the short-axis longitudinal stack, a two-chamber left ventricular (LV) cine CMR image was used to aid in tracking. Four equally spaced parallel short-axis slices were then interpolated from the stacked data corresponding to 25%, 50%, 75%, and 100% distance from apex to base, allowing us to partially compensate for longitudinal through-plane motion. For each slice, six rotations around the kinematic axis corresponding to 30°, 90°, 150°, 210°, 270°, and 330° were also tracked corresponding roughly to the conventional long axis (30°, 210°), two-chamber (90°, 270°) and four-chamber (150°, 330°) views [41]. Each slice and rotation correspond to a structured set of 48 points as shown (Fig. 1) with 12 points in each of 4 short-axis slices representing the LV. Each point is tracked throughout the cardiac cycle and splines are used to interpolate myocardial position between points. During tracking, 1:2 spatial averaging of pixel intensities within the slices in the longitudinal axis allowed us to better visualize myocardial motion in the long axis plane.

Additionally, due to respiratory artifacts, some slices were not aligned along the longitudinal axis. To account for this shifting, and after tracing wall motion in the short-axis, manual correction was applied in the long axis view by using all other slices within the volume for context. When all of the corresponding points were defined, splines were used to interpolate a dynamic 3D mesh with each boundary (endocardial and epicardial) sampled uniformly at 60 interpolated time points across one cardiac cycle, 60 rotations around the longitudinal axis, and 60 slices from base to apex as described previously [25]. We then used these contour maps to obtain quantitative measurements of localized cardiac kinematics (Fig. 1F–I) such as Green–Lagrange circumferential strain (E_cc_; Additional file 1), longitudinal strain (E_ll_; Additional file 2), radial strain (E_rr_; Additional file 3), and surface area strain (E_a_; Additional file 4). We also determined the systolic strain rate for each strain quantity and corresponding region across a normalized cardiac cycle as described previously [40]. Additional metrics such as localized early diastolic strain rate and late diastolic strain rate comparing the entire DMD CMP cohort (n = 43) to a healthy control group (n = 25) were also collected for comparison (Additional files 5, 6). After obtaining these parameters, we mapped the kinematic changes in a localized manner across a cardiac cycle, comparing patient data sets using regions defined by the American Heart Association (AHA) 17-segment model [42].

### Localized cardiac strain derivation

**Fig. 2:**
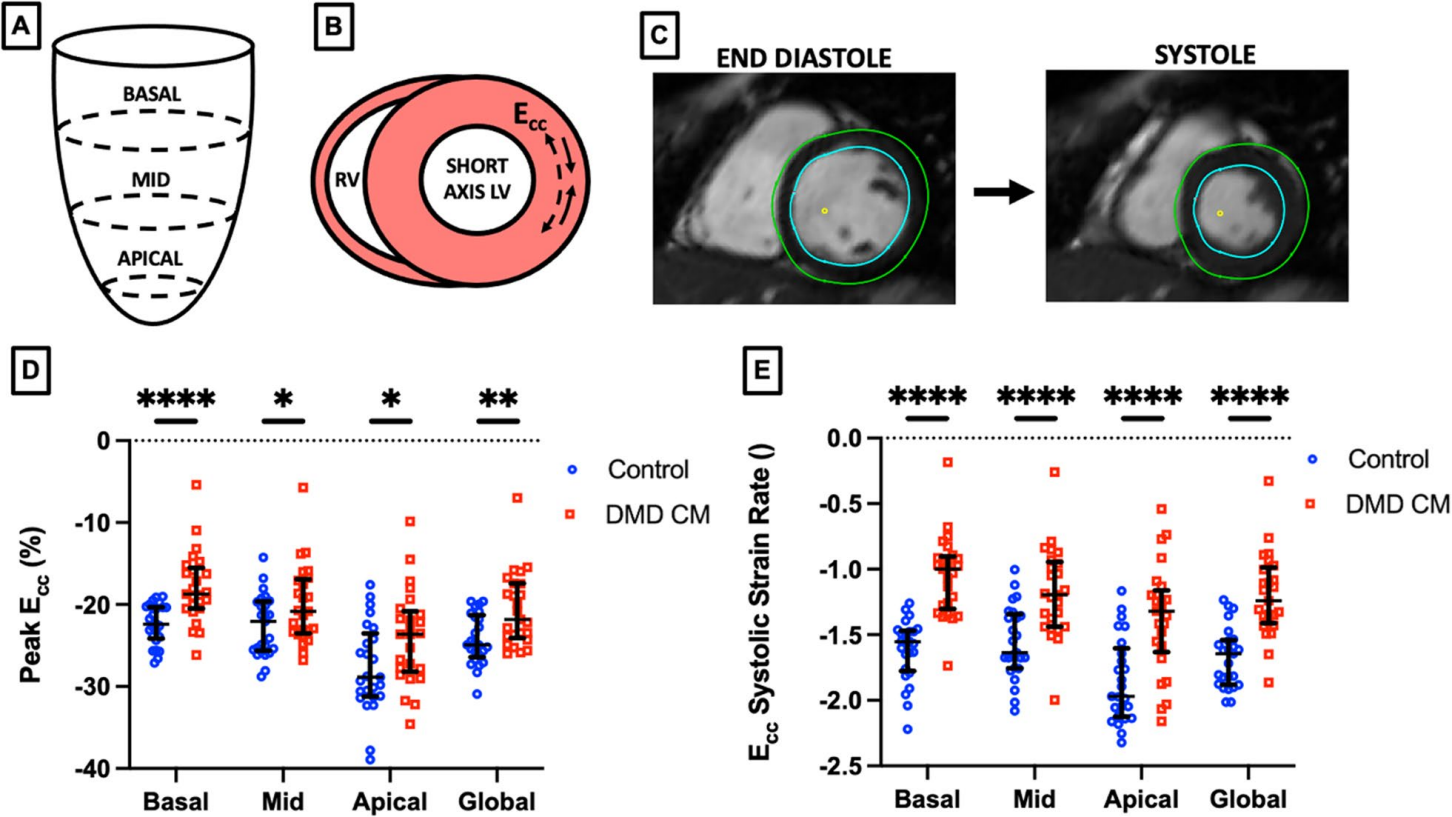
3D + time CMR- derived localized E_cc_ peak circumferential strain and systolic strain rate can discriminate between DMD (n = 43) and healthy control (n = 25) subjects. **A**, **B** Schematic representation of regional circumferential strain (E_cc_). **C** Example CMR image slice depicting feature-tracked endocardium (blue) and epicardium (green) **D** Localized peak E_cc_ and **E** systolic strain rate (normalized to cardiac cycle) shows significant differences between healthy control subjects and DMD patients- particularly in the basal region for peak strain and in all regions for systolic strain rate. *p < 0.05, **p < 0.01, ****p < 0.001, error bars depict median and interquartile range

To estimate circumferential strain (E_cc_), we calculated the circumferential component of the Green Lagrange strain tensor from the 3D + time mesh at each short-axis slice location [40, 43]:

1 $${E}_{cc}\left(z,t\right)=\frac{1}{2}\left[{\left(\frac{C\left(z,t\right)}{{C}_{D}\left(z\right)}\right)}^{2}-1\right]\times 100\%$$

where $C$ represents the relative circumference along a short-axis slice orthogonal to the longitudinal direction $z$ over time $t$ in the cardiac cycle. ${C}_{D}$ is the circumference at end-diastole (i.e. time $t=0$). To obtain localized E_cc_ estimates in the basal, mid-ventricular, and apical regions of the LV, we generated a strain curve over the course of a cardiac cycle at slices corresponding to each slice level (Fig. 2A). Peak strain, systolic strain rate, early diastolic strain rate, and late diastolic strain rates were then extracted from each curve as described previously [40]. Global peak E_cc_ and strain rate were estimated by taking the average values from each region.

Longitudinal strain (E_ll_) was estimated using the engineering small strain approximation in the Lagrangian reference frame:

2 $${E}_{ll}(\theta ,t)=\left[\frac{L\left(\theta ,t\right)-{L}_{D}(\theta )}{{L}_{D}(\theta )}\right]\times 100\%$$

where $L$ represents the length from apex to base along the circumferential boundary at a rotation $\theta$ at time $t$ within the cardiac cycle. ${L}_{D}$ represents the length at end-diastole. Localized peak strain, systolic strain rate, early diastolic strain rate, and late diastolic strain rate were similarly derived from strain curves corresponding to the anterior free wall, anterior, anterior septum, posterior septum, posterior, and posterior free wall sections [40]. Global peak E_ll_ and E_ll_ strain rate were calculated by taking the average of each region.

Radial strain (E_rr_) was also calculated using the engineering small strain approximation in the Lagrangian reference frame:

3 $${E}_{rr}(z,\theta ,t)=\left[\frac{R\left(z,\theta ,t\right)-{R}_{D}(z,\theta )}{{R}_{D}(z,\theta )}\right]\times 100\%$$

where $R$ represents the radial distance between the endocardial boundary and epicardial boundary at a specific longitudinal slice location $z$ and rotational location $\theta$ at time $t$ relative to its corresponding radial distance at end-diastole ${R}_{D}$. Localized E_rr_ peak strain, systolic strain rate, early diastolic strain rate, and late diastolic strain rate values at the basal, mid-ventricular, and apical regions were determined by calculating an average metric from within each slice-level region or alternatively calculated by regions defined by the 17-segment AHA model [42]. Global E_rr_ and E_rr_ strain rate was determined by averaging each region or slice level at peak systole.

Surface area strain (E_a_) was calculated using a similar approximation also in the Lagrangian reference frame:

4 $${E}_{a}(z,\theta ,t)=\left[\frac{A\left(z,\theta ,t\right)-{A}_{D}(z,\theta )}{{A}_{D}(z,\theta )}\right]\times 100\%$$

where $A$ represents the surface area on the endocardial surface between two sequential slice locations along the longitudinal axis $z$ and rotational location $\theta$. As described above, the 3D mesh boundary is sampled uniformly at 60 interpolated time points across one cardiac cycle, with 60 rotations around the longitudinal axis, and 60 slices from base to apex for a total of 3600 nodes at each timepoint [25]. The E_a_ at each timepoint therefore measures the surface area changes corresponding to one of the nodes compared to its surface area at end-diastole ${A}_{D}$. Localized E_a_ peak strain, systolic strain rate, early diastolic strain rate, and late diastolic strain rate values at the basal, mid-ventricular, and apical regions were determined by calculating an average metric from all nodes within each slice level or alternatively in each region defined by the 17-segment model. Global E_a_ was determined by averaging all corresponding nodes at peak systole.

### Statistical analysis

We performed statistical assessment using Prism 9 (GraphPad Software, San Diego, California, USA). Normal distribution was assessed for each metric using an Anderson–Darling test (*p* < 0.05) and non-parametric statistics were used for data sets not following a normal distribution. Unpaired t-tests for normally distributed data comparison or Mann–Whitney tests were used to determine statistical differences between DMD CMP and control groups. All cohort-specific metrics are reported as median [interquartile range] or mean ± standard deviation. We also report area under the curve (AUC) measurements derived from receiver operator characteristic analysis as a measure of sensitivity and specificity of each metric for distinguishing between control and DMD CMP groups. Spearman’s rho was used to determine statistical correlation with *p* < 0.05 indicating significance.

## Results

### Study population

For this study, we analyzed CMR images from 43 DMD CMP patients (median age: 12.23 yrs [10.6–16.5]; [interquartile rnage]) and 25 male healthy controls (median age: 16.2 yrs [13.3–20.7]). Control subjects were healthy volunteers, 12 (48%) of which were imaged using gadolinium contrast. All DMD CMP patients were imaged with gadolinium contrast (n = 43, 100%). In the DMD CMP group there was a range of CMP severity: 15 (35% of total) had LV ejection fraction (LVEF) > 55% with no findings of myocardial LGE, 15 (35% of total) had findings of LGE with LVEF > 55% and 13 (30% of total) had LGE with LVEF < 55% (DMD CMP Groups A, B, and C respectively, Table 1).

**Table 1:** Control and Duchenne muscular dystrophy-associated cardiomyopathy (DMD CMP) patient information

	Control	DMD CMP	DMD CMP subgroups			
			DMD CMP Age Matched	DMD CMP Group A	DMD CMP Group B	DMD CMP Group C
	n = 25	n = 43	n = 25	n = 15	n = 15	n = 13
Age (years)	16.2 [13.3–20.7]	12.2 [10.7–16.5]*	15.7 [14.0–17.8]	10.6 [8.7–11.7]*	12.9 [11.2–16.0]*	17.4 [ 14.0–19.1]
Height (cm)	170 [160–185]	147 [132–160]*	158 [152–168]*	127 [122–142.5]*	152 [145–157.75]*	163 [145–170]*
Weight (kg)	67.0 [54.9–81.4]	49.1 [37.5–65.2]*	58.2 [49.5–72.0]	38.6 [31.9–57.8]*	52.3 [45.9–62.1]	55.7 [42.7–64.5]
LVEF (%)	61.0 [57.8–64.3]	59.0 [53.0 – 62.0]*	56.0 [49.0 – 60.0]*	62.0 [59.5 – 64.0]	59.0 [58.5–60.5]	49.0 [47.0 – 52.0]*
LVEF < 55%, n (%)	0 (0)	13 (30.23)	11 (44)	0 (0)	0 (0)	13 (100)
LVEDVI (%)	82 [73–90]	60 [55–73]*	60 [56–73]*	57 [53–66]*	59 [56–62]*	73 [68–83]
LVESVI (%)	55 [40–69]	25 [22–35]*	25 [23–37]	22 [19–25]*	25 [23–26]*	37 [34–50]*
LV CO (L/min)	5.5 [5.1–7.1]†	4.9 [4.1–5.9]*	5.2 [4.2–6.2]	4.5 [3.9–5.7]*	4.9 [ 4.4–5.7]*	5.2 [4.6–6.2]
+ LGE, n (%)	0 (0)†	28 (65)	20 (80)	0 (0)	15 (100)	13 (100)
Systolic BP (mmHg)	125 [112–132]†	114 [107 -118]	114 [107–118]	116 [107–122]	115 [112–115.5]	108 [103–115]
Diastolic BP (mmHg)	57 [54–57]†	66 [63–74]*	68 [63–75]*	64 [63–72]	70 [65–75]*	66 [60–69]
HR (BPM)	75 [68–91]†	99 [92–110]*	98 [84–110]*	102 [96–118]*	98 [89–104]*	101 [90–109]
BSA (m^2^)	1.74 [1.60–1.99]†	1.45 [1.19–1.68]*	1.58 [1.46–1.84]	1.19 [1.07–1.465]*	1.50 [1.37–1.66]*	1.50 [1.43–1.75]

DMD CMP, Duchenne muscular dystrophy-associated cardiomyopathy; LVEF, left ventricular ejection fraction; LVEDVI, left ventricular end diastolic volume indexed to body surface area (BSA); LVESVI, left ventricular end systolic volume indexed to BSA; LV CO, left ventricular cardiac output; LGE, late gadolinium enhancement. Values reported as median [interquartile range] or as number of patients (percentage of group)—n (%)

DMD CMP Group A: -LGE, LVEF > 55 (n = 15), DMD CMP Group B: + LGE, LVEF > 55 (n = 15), DMD CMP Group C: + LGE, LVEF < 55 (n = 13) †some datapoints from full cohort unavailable, *p < 0.05 compared to control group

### Circumferential strain

Figure 2 demonstrates the ventricular levels from which we derived E_cc_ metrics. Peak basal E_cc_ showed the strongest significant difference between control subjects and DMD CMP patients (*p* < 0.001, AUC = 0.83). Mid-ventricular, apical, and global peak E_cc_ showed more modest differences with values summarized in Table 2. The differences in the basal region compared to the other regions suggests more significant CMP involvement at the base of the LV. This is consistent with other literature [44] and the LGE distribution pattern, which is more prevalent in the free wall of the basal and mid-LV slices (Additional file 7). E_cc_ systolic strain rate also had the greatest difference between DMD CMP and control subjects at the base (*p* < 0.001, AUC = 0.95), though mid-ventricular, apical, and global strain were also highly significant. As might be expected, regional strain parameters are moderately correlated with one another (Additional file 8).

**Table 2:** Localized peak strain and systolic strain rate derived from 3D + time CMR

Strain	Region	Peak Strain Value				Systolic Strain Rate			
		Control (n = 25)	DMD CMP (n = 25)	*p*	AUC	Control (n = 25)	DMD CMP (n = 25)	*p*	AUC
Circumferential (E_cc_)	Basal	− 22.5 ± 2.4	− 17.9 ± 4.3*	0.010	0.83 [0.71–0.95]	− 1.6 ± 0.2	− 1.1 ± 0.1*	< 0.001	0.95 [0.89–1.00]
	Mid	− 22.6 ± 3.8	− 20.0 ± 4.8*	0.452	0.66 [0.51–0.81]	− 1.6 ± 0.3	− 1.3 ± 0.2*	0.001	0.81 [0.69–0.93]
	Apical	− 27.3 ± 5.1	− 24.0 ± 5.7*	0.054	0.68 [0.53–0.83]	− 1.9 ± 0.3	− 1.6 ± 0.4*	< 0.001	0.82 [0.70–0.93]
	Global	− 24.2 ± 3.1	− 20.6 ± 4.5*	0.080	0.75 [0.61–0.88]	− 1.7 ± 0.2	− 1.3 ± 0.2*	< 0.001	0.89 [0.80–0.98]
Longitudinal (E_ll_)	Anterior FW	− 11.2 ± 2.4	− 10.3 ± 2.8	0.998	0.55 [0.39–0.71]	− 0.7 ± 0.2	− 0.6 ± 0.1*	0.174	0.7 [0.56–0.85]
	Anterior	− 10.8 ± 2.2	− 10.3 ± 2.9	0.999	0.53 [0.36–0.69]	− 0.7 ± 0.1	− 0.6 ± 0.1*	0.256	0.69 [0.55–0.84]
	Anterior S	− 9.6 ± 2.1	− 9.0 ± 2.9	0.999	0.57 [0.40–0.73]	− 0.6 ± 0.2	− 0.5 ± 0.1	0.799	0.66 [0.50–0.81]
	Posterior S	− 9.7 ± 2.5	− 9.1 ± 2.5	0.999	0.6 [0.44–0.76]	− 0.6 ± 0.2	− 0.5 ± 0.1	0.875	0.64 [0.48–0.80]
	Posterior	− 11.8 ± 2.3	− 10.2 ± 3.2	0.655	0.66 [0.51–0.82]	− 0.7 ± 0.2	− 0.6 ± 0.1*	0.058	0.72 [0.57–0.86]
	Posterior FW	− 11.5 ± 2.6	− 10.1 ± 2.9	0.753	0.64 [0.49–0.80]	− 0.7 ± 0.2	− 0.6 ± 0.1*	0.019	0.79 [0.66–0.92]
	Global	− 10.8 ± 1.8	− 9.8 ± 2.6	0.995	0.61 [0.45 − 0.77]	− 0.7 ± 0.1	− 0.6 ± 0.1*	0.262	0.73 [0.59–0.88]
Radial (E_rr_)	Basal	41.9 ± 12.8	21.1 ± 12.0*	< 0.001	0.88 [0.79–0.97]	2.8 ± 1.1	0.8 ± 0.8*	< 0.001	0.88 [0.79–0.97]
	Mid	33.6 ± 13.7	19.4 ± 11.1*	0.001	0.79 [0.67–0.91]	2.3 ± 0.9	0.9 ± 0.8*	< 0.001	0.83 [0.72–0.94]
	Apical	25.4 ± 12.3	15.3 ± 10.2*	0.054	0.75 [0.62–0.89]	1.4 ± 0.7	0.5 ± 0.7*	0.052	0.8 [0.68–0.92]
	Global	33.7 ± 11.4	18.7 ± 10.3*	< 0.001	0.84 [0.73–0.95]	2.2 ± 0.8	0.8 ± 0.7*	< 0.001	0.88 [0.79–0.97]
Surface Area (E_a_)	Basal	− 31.6 ± 3.2	− 25.4 ± 5.2*	0.009	0.85 [0.74–0.96]	− 2.2 ± 0.3	− 1.5 ± 0.2*	< 0.001	0.96 [0.90–1.0]
	Mid	− 31.3 ± 4.7	− 27.3 ± 6.3*	0.293	0.68 [0.53–0.83]	− 2.1 ± 0.3	− 1.7 ± 0.2*	< 0.001	0.84 [0.73–0.95]
	Apical	− 38.6 ± 6.8	− 35.1 ± 8.6	0.232	0.62 [0.47–0.78]	− 2.7 ± 0.5	− 2.3 ± 0.5*	< 0.001	0.79 [0.66–0.91]
	Global	− 33.6 ± 4.1	− 29.2 ± 6.2*	0.106	0.72 [0.58–0.86]	− 2.3 ± 0.3	− 1.8 ± 0.2*	< 0.001	0.9 [0.82–0.99]

FW, free-wall; S, septal; DMD, CMP Duchenne muscular dystrophy-associated cardiomyopathy; AUC, area under the curve. *p < 0.05 (Tukey multiple comparisons adjusted)

### Longitudinal strain

**Fig. 3:**
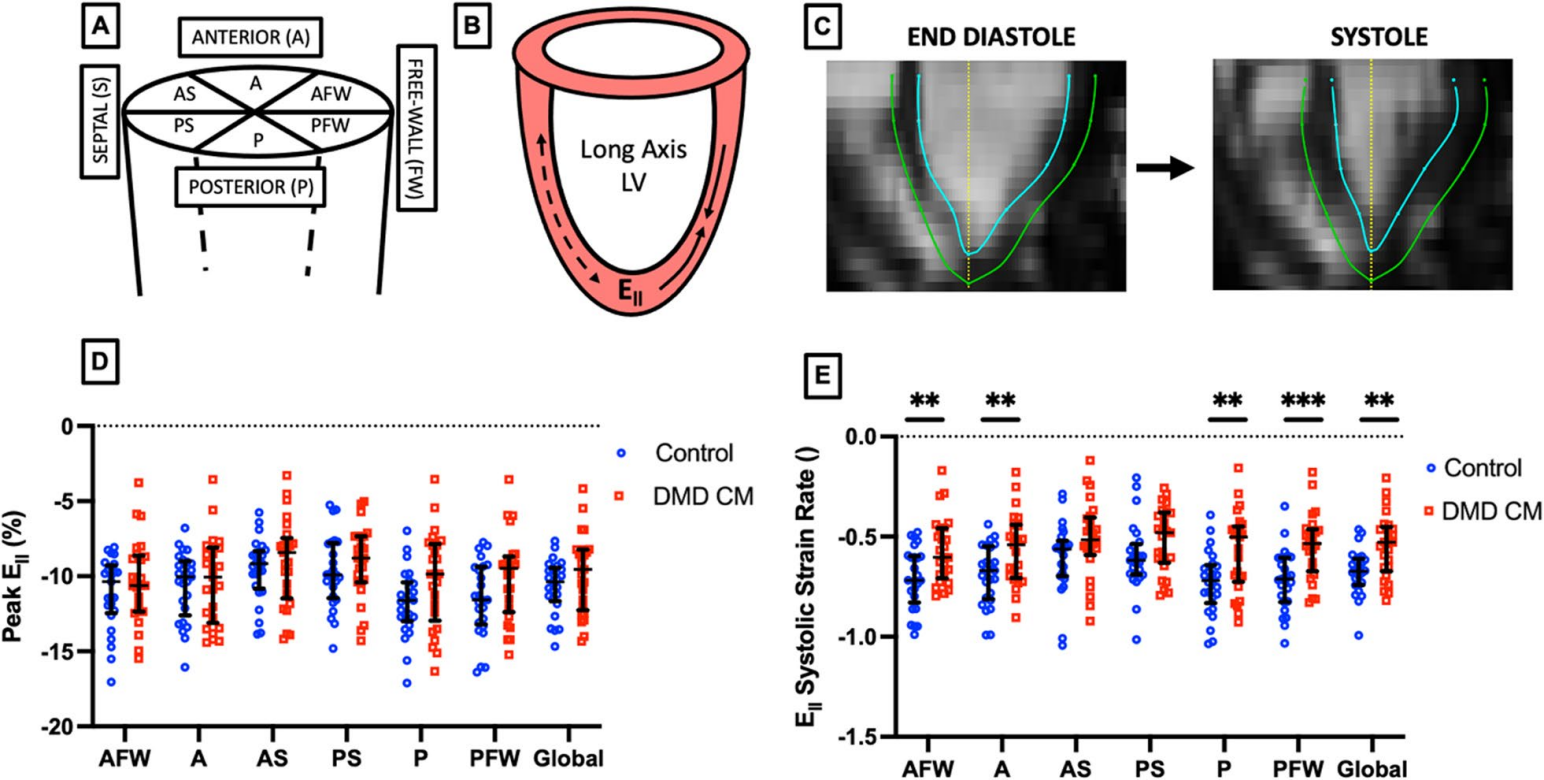
3D + time CMR-derived localized systolic strain rate can discriminate between DMD patients (n = 43) and healthy control (n = 25) subjects. **A**, **B** Schematic representation of localized longitudinal strain (E_ll_) and strain rate. **C** Example 4D CMR long-axis image slice depicting feature-tracked endocardium (blue) and epicardium (green), **D** Localized peak E_ll_ between DMD patients and healthy control subjects less significant than peak E_ll_, however **E** E_ll_ systolic strain rate (normalized to cardiac cycle) shows significant differences between healthy control subjects and DMD patients in all regions. **p < 0.01, ***p < 0.001, error bars depict median and interquartile range

We calculated localized peak E_ll_ and E_ll_ systolic strain rate from 3D + time CMR segmentations within six segments distributed around the LV corresponding to the anterior free wall, anterior, anterior septal, posterior septal, posterior, and posterior free-wall regions of the LV (Fig. 3A). We found that peak E_ll_ in DMD CMP patients was not significantly different from that of healthy controls in any region, though our results approached significance in the posterior portion of the LV (*p* = 0.050, AUC = 0.66), with a modest average difference in posterior peak E_ll_ between groups. Interestingly, we did find modest statistically significant differences between DMD CMP and healthy controls in systolic strain rate for all regions of analysis except for the posterior septal and anterior septal regions. We also observed the strongest significant difference in the posterior free-wall region.

### Radial strain

**Fig. 4:**
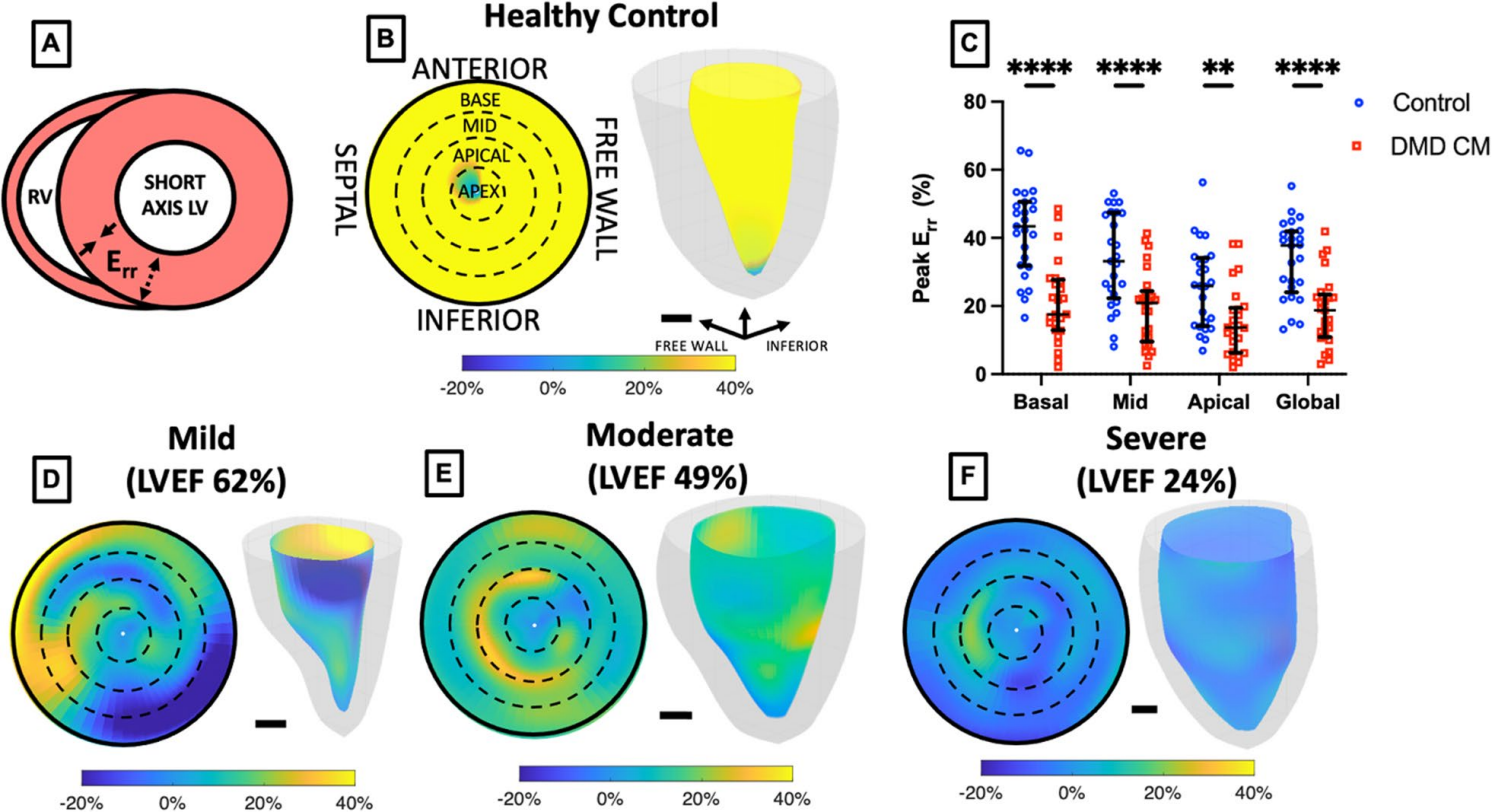
3D + time CMR localized radial strain significantly different in DMD vs. Control patients. **A** Short-axis schematic representation of radial strain (E_rr_). **B** Healthy control polar plot with overlaid slice-level polar map and 3D LV colorized endocardium representation of localized E_rr_. **C** Localized and global peak E_rr_ significantly different between healthy control subjects and DMD patients. **D** Mild, **E** moderate, and **F** severe (as determined by LV ejection fraction) DMD patient examples of 17 segment polar and 3D colorized LV representations of peak E_rr_. Scale bar = 1 cm. **p < 0.01, ****p < 0.001, error bars depict median and interquartile range

Radial strain (E_rr_) from 3D + time CMR was calculated by measuring the relative radial distance change between the endocardial and epicardial boundaries of the derived 3D mesh throughout the cardiac cycle (Fig. 4). We observed highly significant differences between DMD CMP and control groups for peak regional radial strain in the basal, mid-ventricular, apical, and global regions (Fig. 4C). Qualitative maps show stark differences in the strain pattern comparing healthy controls (Fig. 4B) to DMD patients with mild, moderate, and severe CMP (Fig. 4D–F) as determined by LVEF.

E_rr_ strain rate was also significantly different between DMD CMP and healthy controls across all evaluated regions of the myocardium including basal, mid-ventricular, apical, and global regions. Notably, we see again that the regional basal strain metric for E_rr_ and E_rr_ strain rate have the highest AUC values among other regions of the myocardium. We also see that E_rr_ strain rate values in our cohort, in general, can differentiate DMD CMP vs. control subjects more effectively than peak strain values alone.

### Surface area strain

**Fig. 5:**
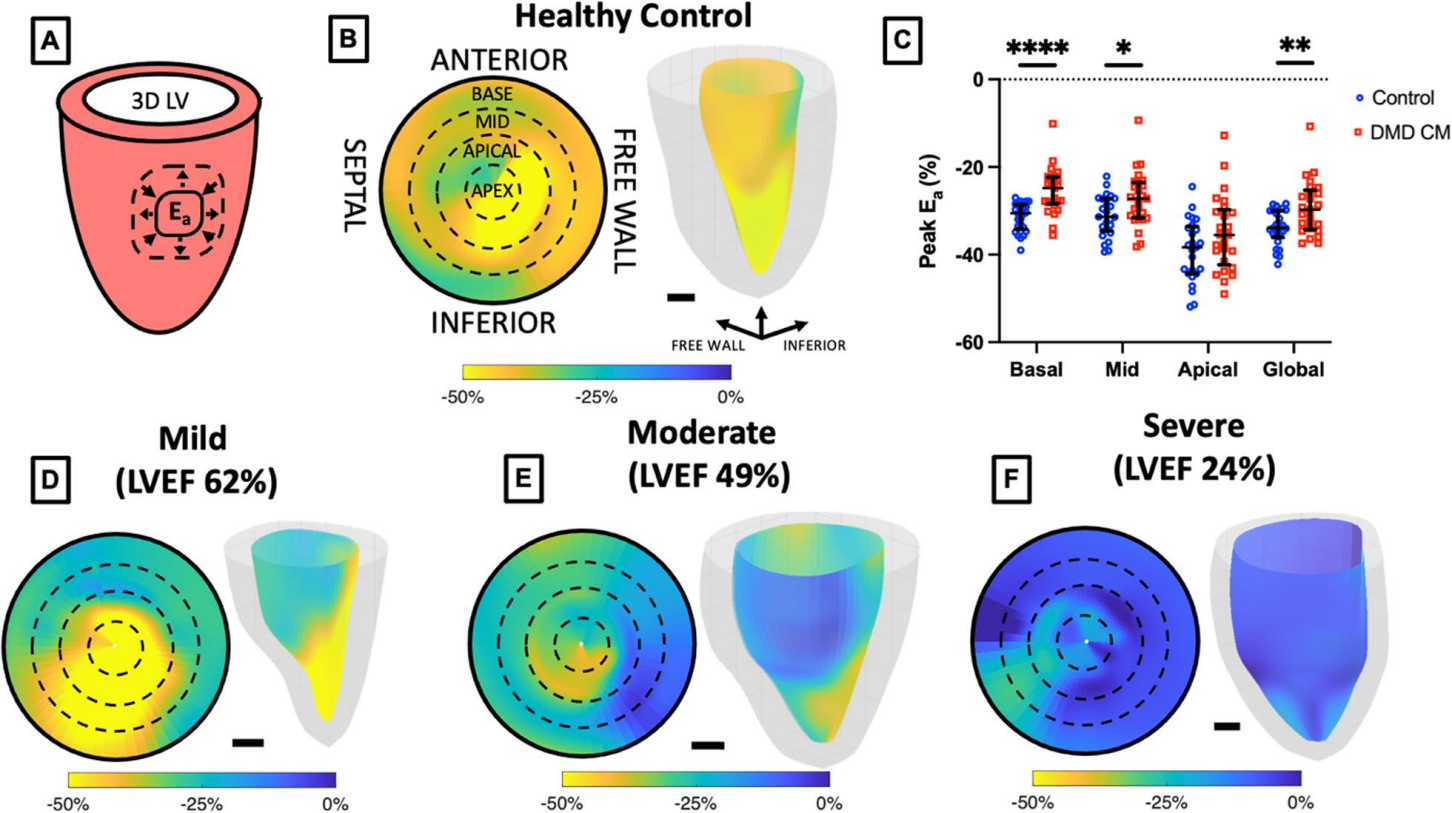
3D + time CMR localized surface area strain significantly different in DMD vs. healthy control subjects. **A** Short-axis schematic representation of surface area strain (E_a_) **B** Healthy control polar plot with overlaid slice-level polar map and 3D LV colorized endocardium representation of local E_a_. **C** Localized and global peak E_a_ significantly different between healthy control subjects and DMD patients. **D** Mild, **E** moderate, and **F** severe (as determined by LVEF) DMD patient examples of 17 segment polar and 3D colorized LV representations of peak E_a_. Scalebar = 1 cm. *p < 0.05, **p < 0.01, ****p < 0.001, error bars depict median and interquartile range

**Fig. 6:**
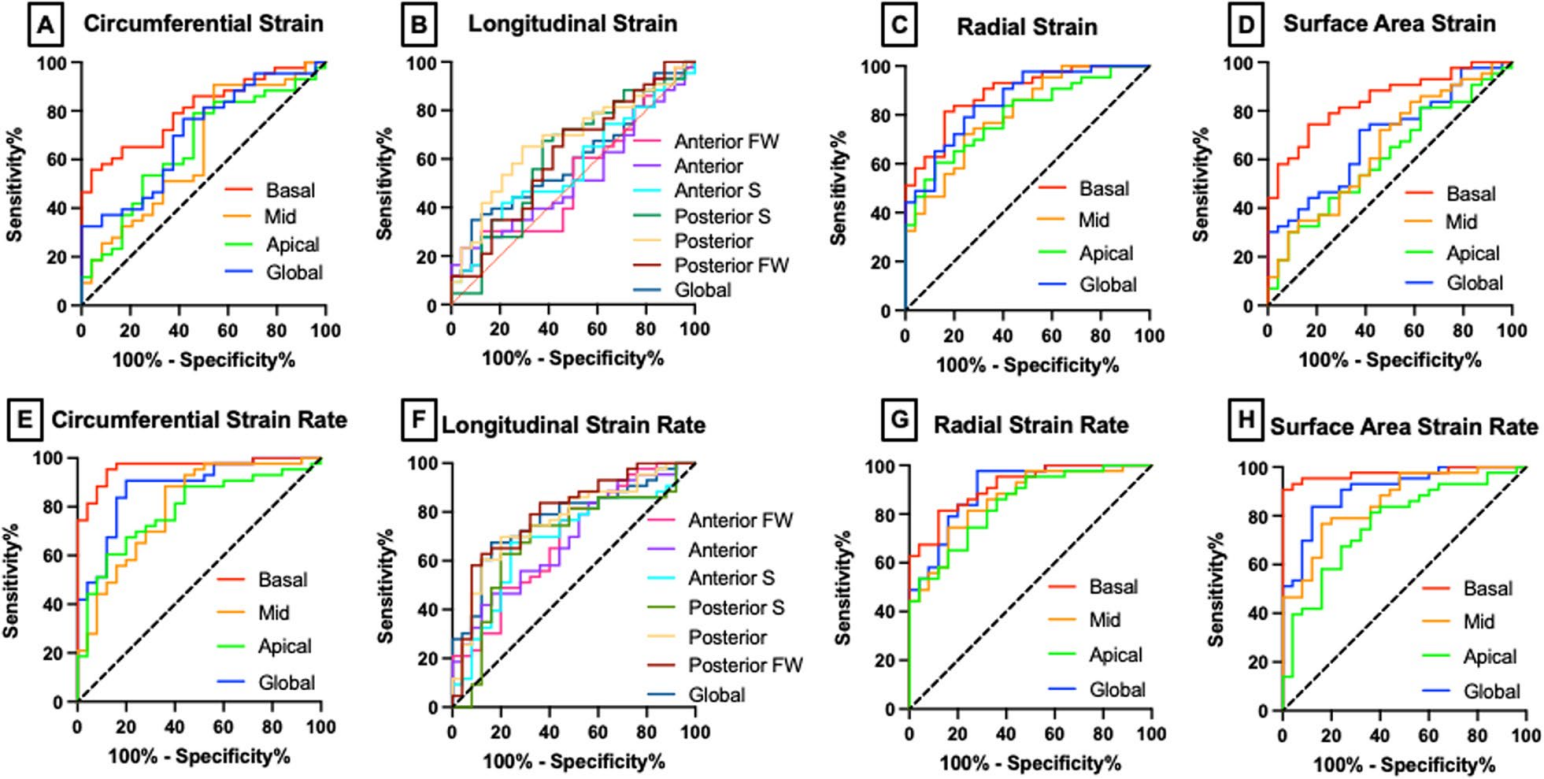
3D + time CMR-derived localized strain and strain rate comparison for discriminating DMD cardiomyopathy (CMP) (n = 43) vs. healthy controls (n = 25) subjects using Wilson/Brown method for receiver operator characteristic curve analysis

Surface area strain (E_a_) is a metric unique to 3D imaging in that it is a measure of the surface area change on the surface of the myocardium over the course of a cardiac cycle (Fig. 5A). Peak E_a_ measured on the endocardium at peak systole shows striking qualitative differences between healthy controls (Fig. 5B) and DMD patients with mild, moderate, and severe CMP (Fig. 5D–F) as measured by LVEF. Here again, we see characteristic patterns of low magnitude strain in the basal and free wall regions similar to the pattern of LGE demonstrated in this cohort (Additional file 7). Following this pattern, we noted a highly significant difference in peak E_a_ between healthy controls and DMD CMP groups in the basal region (Fig. 6) and a modest difference in the mid-ventricular region. No difference was found in the apical region, though there was a difference for peak global E_a_ (results summarized in Table 2).

E_a_ systolic strain rate was significantly different between healthy controls and DMD CMP patients in all analyzed regions, though this difference again appeared to be strongest in the basal region (*p* < 0.001, AUC = 0.96). In fact, this difference appeared to be the strongest found in this study, followed closely by basal E_cc_ systolic strain rate (*p* < 0.001, AUC = 0.95). These strain quantities may both be a measure of pathologic changes to circumferentially oriented fibers. E_a_ systolic strain rate values along other regions of the heart were also significantly different between healthy controls and DMD CMP with mid-ventricular apical, and global all showing stronger changes than regional peak E_a_ alone.

### Regional strain metrics differentiate mild and severe cardiomyopathy

**Fig. 7:**
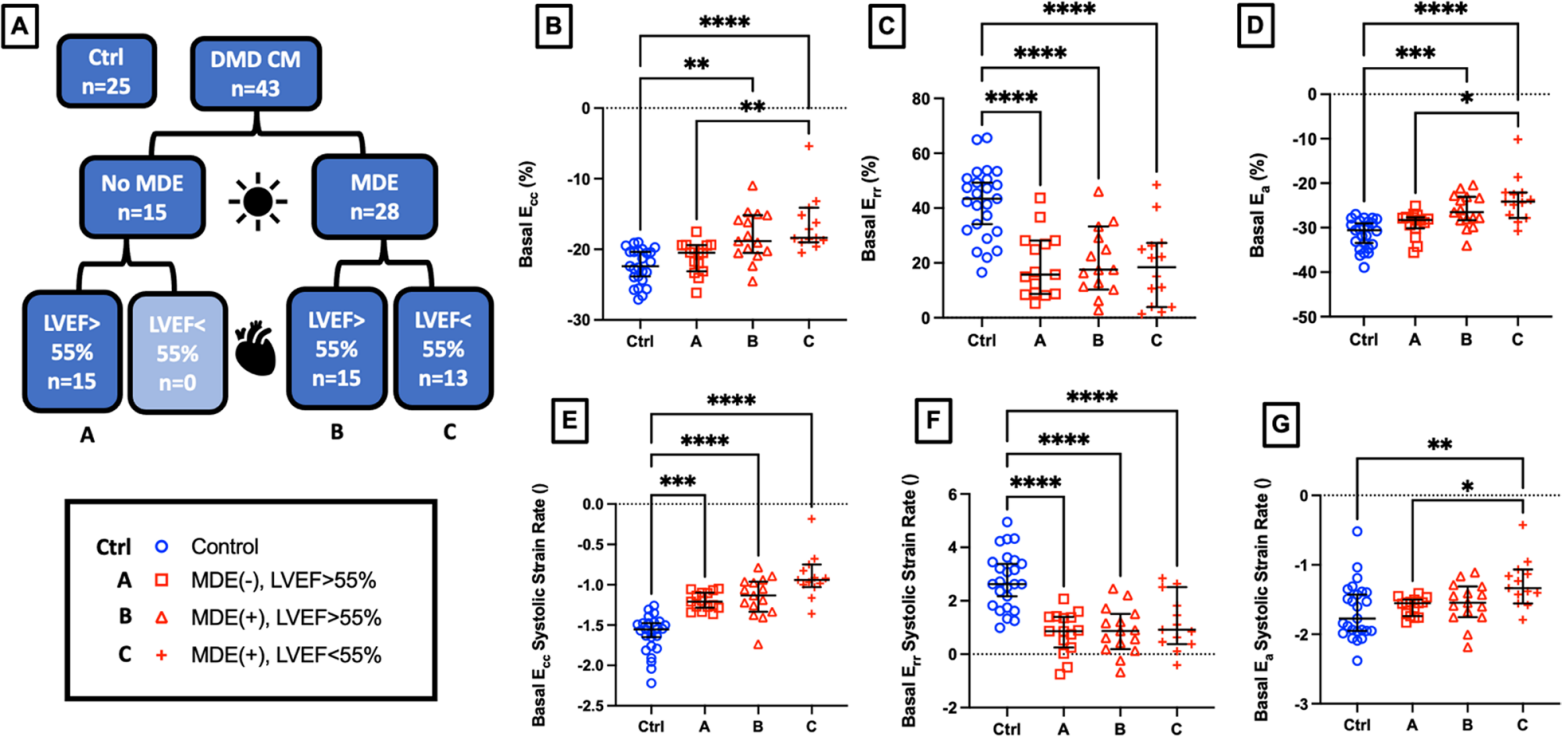
Regional strain differentiates mild, moderate, and severe DMD CMP from healthy control subjects. **A** Stratification paradigm for healthy controls (n = 25) and DMD CMP (n = 43) patients between those without myocardial delayed enhancment (MDE) and an LVEF >55% (Group A, n = 15) those with MDE and LVEF > 55% (Group B, n = 15) and those with MDE and LVEF < 55% (Group C, n = 13). B–D) Basal region peak circumferential strain (E_cc_; **B**), radial strain (E_rr_; **C**), and surface area strain (E_a_; **D**) shows differences between healthy controls and DMD CMP groups A, B, and C. Basal region systolic strain rates for E_cc_ (**E**), E_rr_ (**F**), and E_a_ (**G**), show significant differences between healthy controls and DMD CM groups A, B, and C. *p < 0.05, **p < 0.01,***p < 0.001 ****p < 0.001, error bars depict median and interquartile range

We differentiated the full cohort of DMD CMP patients (n = 43) into those without LGE findings and LVEF > 55% (Group A, n = 15), those with LGE and LVEF > 55% (Group B, n = 15), and those with LGE and LVEF < 55% (Group C, n = 13; Fig. 7A). Within the DMD CMP subgroups, basal peak E_cc_, basal peak E_a_, and basal E_a_ systolic strain rate metrics also demonstrated significant group differences between DMD CMP groups A and C (*p* = 0.004, p = 0.039, *p* = 0.046 respectively). Basal peak E_cc_ (Fig. 7B) revealed significantly decreased strain magnitudes between healthy controls and DMD CMP groups B (*p* = 0.003) and C (*p* < 0.001), but not group A (*p* = 0.604). However, basal systolic strain rate magnitude was significantly decreased for DMD CMP groups A, B, and C (p < 0.001 for all) compared to healthy controls (Fig. 7E). Basal peak E_rr_ showed significant differences between healthy controls and DMD CMP groups A, B, and C (*p* < 0.001 for all). Similar differences were also noted in basal E_rr_ systolic strain rate (A, B, C, p < 0.001 for all; Fig. 7C, F). Basal peak E_a_ showed differences between healthy controls and DMD CMP groups B (p < 0.001) and C (*p* < 0.001) but not A (*p* = 0.260) while E_a_ systolic strain rate showed significant differences between control and DMD CMP group C only (*p* = 0.004), but not for group A (*p* = 0.930) or B (*p* = 0.722).

### 3D + time imaging kinematics correlate with functional and compositional data

Localized strain values derived from 3D + time CMR in DMD CMP patients (n = 43) correlated with both functional and compositional characteristics from CMR. With respect to LVEF, slice-level peak E_cc_ values were correlated among all assessed regions including basal, mid-ventricular, apical, and global regions. Peak E_cc_ values were also correlated with slice-level ECV in the basal, mid-ventricular, apical, and global, regions. Similar patterns of correlation with LVEF and ECV were observed for E_cc_ systolic strain rate, peak E_a_, and E_a_ systolic strain rate summarized in Table 3. Localized T1 was significantly correlated with peak basal E_a_ and with the basal strain rate for E_cc_, E_rr_, and E_a_. Insignificant to modest correlation was observed between slice-level strain values and global T1 and T2 values. Modest or no significant correlations with LVEF, T1, T2, or ECV were observed between peak E_ll_, E_ll_ strain rate, E_rr_, and E_rr_ strain rate in the mid-ventricular and apical regions. Unsurprisingly, moderate correlations were also found between peak strain and systolic strain rate (Additional file 9) suggesting these parameters, derived from the same strain curve, might be similarly useful when correlating values with functional metrics.

**Table 3:** Functional correlation of localized strain parameters

Strain	Region	Peak strain correlation				Strain rate correlation			
		LVEF	T2	T1	ECV	LVEF	T2	T1	ECV
Circumferential (E_cc_)	Basal	− 0.50*	0.30	0.28	0.51*	− 0.51*	0.28	0.33	0.58*
	Mid	− 0.46*	0.35	0.01	0.42*	− 0.42*	0.21	− 0.07	0.41*
	Apical	− 0.43*	0.15	− 0.01	0.46*	− 0.40*	0.15	0.13	0.51*
	Global	− 0.53*	0.37	0.06	0.51*	− 0.44*	0.22	− 0.04	0.55*
Longitudinal (E_ll_)	Anterior FW	− 0.32	0.24	0.14	0.34	− 0.23	0.24	0.15	0.43*
	Anterior	− 0.02	0.05	0.03	0.19	− 0.15	0.04	0.06	0.27
	Anterior S	− 0.20	0.13	0.06	0.24	− 0.14	0.08	0.17	0.28
	Posterior S	− 0.18	0.14	0.16	0.19	− 0.19	0.20	0.13	0.25
	Posterior	− 0.22	0.14	0.05	0.26	− 0.18	0.04	0.02	0.25
	Posterior FW	− 0.31	0.23	0.05	0.33	− 0.27	0.23	0.01	0.35
	Global	− 0.23	0.16	0.11	0.31	− 0.22	0.13	0.13	0.36
Radial (E_rr_)	Basal	− 0.13	− 0.19	− 0.18	− 0.17	− 0.15	− 0.22	− 0.40*	− 0.12
	Mid	− 0.15	− 0.11	− 0.06	− 0.17	− 0.07	− 0.10	− 0.21	− 0.26
	Apical	− 0.22	0.01	0.01	0.09	− 0.09	− 0.07	− 0.10	− 0.04
	Global	− 0.16	− 0.09	− 0.04	− 0.13	− 0.14	− 0.05	− 0.25	− 0.14
Surface Area (E_a_)	Basal	− 0.45*	0.24	0.30	0.40*	− 0.45*	0.29	0.44*	0.56*
	Mid	− 0.46*	0.30	0.05	0.46*	− 0.46*	0.22	0.07	0.50
	Apical	− 0.42*	0.15	0.02	0.47*	− 0.35	0.11	− 0.12	0.44*
	Global	− 0.50*	0.32	0.11	0.51*	− 0.46*	0.21	0.11	0.56*

FW, free-wall; S, septal; LVEF, left ventricular ejection fraction; ECV, extracellular volume Spearman r used for correlation. *p < 0.05 (Bonferroni–corrected)

## Discussion

This study describes the first use of a novel method for 3D cine CMR feature-tracking (FT) strain analysis using sequentially stacked 2-dimensional cine CMR images. This study also demonstrates the utility of this method in patients with DMD CMP. We showed that localized strain metrics derived from this 4D image analysis are both sensitive and specific for characterizing DMD CMP disease severity. To our knowledge, this is the first report that localized surface area strain and strain rate metrics derived from 3D cine CMR were used to characterize CMP in a DMD cohort. We observed that basal E_a_, E_cc_, and E_rr_ peak strain and strain rate were the most sensitive and specific metrics for differentiating DMD CMP from healthy control subjects. In general, we found that for every strain metric assessed, slice-level localized strain rate was better able to differentiate between DMD and control subjects compared to corresponding peak strain alone. Basal E_a_ systolic strain rate had the best differentiation with an AUC of 0.96. Basal E_rr_, basal E_rr_ systolic strain rate and basal E_cc_ systolic strain rate magnitude values were significantly decreased in mild cardiomyopathy (LGE-, LVEF > 55%) compared to a healthy control group. Localized E_a_ and E_cc_ peak strain and strain rate metrics also had the strongest correlation with LVEF, T1, and ECV values while E_rr_ and E_ll_ were less strongly correlated with LVEF, T1, and ECV. These data suggest that localized and kinematic analysis of 3D cine CMR images in DMD patients may provide a more robust analysis for assessing CMP than global or peak strain values alone.

Patients with DMD universally develop cardiomyopathy [45]. However, the age of onset, the time course, and the severity of CMP are highly variable necessitating more refined measures for assessing CMP severity [14, 15]. Although LGE is present as early as 7 years of age it is only apparent when a significant amount of damage has occurred. Traditional functional assessment by LVEF is limited as it is only abnormal in later stages of disease when the process may no longer be reversible. As such, developing early markers of disease can shift the treatment paradigm from rescue to prevention. These early abnormalities provide novel biomarkers and surrogate outcome measures of disease progression.

In this study, we observed the most significant differences, largest AUC values, and strongest correlation to LVEF in basal circumferential and basal E_a_ when comparing DMD patients to healthy controls. The results demonstrate the value of E_cc_ as an imaging biomarker in DMD and are consistent with other studies [46, 47]. In a recent study, Siddiqui et al. also showed 3D CMR-derived E_cc_ was better able to predict the onset of DMD CMP than conventional 2D CMR-derived strain values [48]. Similar to our technique, Siddiqui et al. accomplished 3D FT in DMD cardiomyopathy using 3D interpolation of the endocardial and epicardial boundaries from 2D slices. This 3D FT strain technique has been shown to have superior reproducibility compared to 2D FT in CMR and has been well described by Liu et al. [49].

To our knowledge, no previous studies have explored the role of strain rate derived from 3D cine CMR in a population of patients with DMD-associated CMP. Strain rate has been a predominantly echocardiographic-based measure likely due to its superior temporal resolution (< 5 ms) compared to CMR (~ 20–50 ms) [50]. Additionally, strain rate measurements that use tagging may be less reproducible due to tag fading [51]. In the CMR FT technique we present here, we do not have issues with tag fading which provides an advantage for strain rate estimation. In addition, our method of interpolation between frames allows us to estimate strain rate despite a relatively lower temporal resolution. However, as with all strain and strain rate estimation methods, it should be noted that variability in heartrate will impact the temporal resolution differently for each cardiac cycle analyzed. This variability may affect strain rate estimations. Our strain rate calculation method, like others used for CMR [52] is based on calculating the slope of the strain curve and as such values may be under- or overestimated depending on heartrate. We do note a difference in heartrate between our DMD and healthy control subjects (Table 1), however there is no significant difference between average heartrates between the DMD subgroups we analyzed in this study. One major advantage to our slope calculation and normalization method is that this standardization allows for some added consistency when comparing strain curve slope and shape between patients and groups- even when heartrate is variable within a single patient scan or between multiple scans and patients. These benefits and limitations, however, should be considered when examining the significance of strain rate findings.

In previous studies, strain rate used in assessing myocardial infarction showed good reproducibility using 2D CMR FT [53] and in a healthy control group for both 2D and 3D CMR FT [49]. In this study, we found that for every strain metric assessed, slice-level localized strain rate was better able to differentiate between DMD and healthy control subjects compared to corresponding peak strain alone. These results suggest that valuable information might be missed when only peak strain values are considered. Strain rate differences, particularly those between DMD groups with increasing CMP severity may be an early indication of mechanistic changes in the heart. In myocardial infarction, strain rate has been shown to correlate with regional ischemia and akinetic regions [54, 55]. One explanation for strain rate differences in DMD CMP therefore could be an early manifestation of regional heterogeneity of systolic function that is spatially correlated with regional fibrofatty replacement of healthy myocardium. Further work using animal models or larger clinical datasets may help elucidate a mechanistic explanation for these findings.

Within the group of DMD patients we analyzed (n = 43), we were able to identify three distinct groups- LGE-/LVEF > 55%, LGE + /LVEF > 55%, and LGE + /LVEF < 55%. In our particular cohort of subjects, no patient was observed to be both LGE- and have an LVEF < 55%. Interestingly, we observed that a few of our tested metrics—basal E_rr_, basal E_rr_ systolic strain rate and basal E_cc_ systolic strain rate magnitude values—were significantly decreased in each DMD group compared to a healthy control group. Other metrics including peak basal Ecc, peak basal Ea, and basal Ea systolic strain rate showed significant differences between LGE-/LVEF > 55% and LGE + /LVEF < 55% groups. These results suggest that regional strain metrics derived from 4D CMR may be able to detect early dysfunction even prior to LGE or overt LV dysfunction and differentiate between more mild and severe disease. A comprehensive longitudinal study describing these changes over time in the same patients would be a valuable extension of this work.

While strain rate measurements improved differentiation for every strain quantity we calculated over peak strain alone, we did not observe strong correlations between radial strain and LVEF, T1, T2, or ECV or longitudinal strain and LVEF, T1, T2, or ECV. This may be due in part to the characteristic pattern of DMD associated CMP which primarily affects myofibers in the subepicardial free wall of the LV, though as the condition progresses, transmural fibrosis becomes increasingly more prevalent [44]. Importantly, we also note that we estimate longitudinal strain using the stack of short-axis images with limited resolution in the longitudinal acquisition plane. This low spatial and contrast resolution may contribute to less reliable feature-tracking and E_ll_ estimations. Another reason we may not be observing these correlations is the wider variation of E_ll_ and E_rr_ strain compared to E_cc_, making correlative measures less reliable. A 3D FT CMR analysis in DMD patients done by Siddiqui et al. [48] similar to ours showed insignificant differences in global E_ll_ between DMD and healthy control subjects but did observe differences in global E_rr_ and global E_cc_. This study also reported similar ranges to those we found for E_ll,_ E_cc,_ and E_rr_, derived from 3D FT CMR in DMD CMP. A different meta-analysis examining global longitudinal strain measured by 2D speckle tracking echocardiography in eight studies showed that global longitudinal strain and circumferential strain but not radial strain were significantly decreased in DMD vs healthy subjects, though the study did show heterogeneity in results [56]. As with all strain estimation methods, differences in acquisition modality, method, and analysis should all be considered when interpreting results.

In our analysis we observed significant differences in E_rr_ between healthy and DMD patients, though relatively lower AUC values compared to E_cc_ and E_a_. This may be due in part to the wide range of E_rr_ values in our method. This variability could be due to dyskinesia resulting in a shift of time of contraction or pathological issues related to radial thickening seen in the DMD patients. It could also be a tracking issue due to through-plane motion from circumferential and longitudinal deformation exacerbated by thinner myocardial walls in more advanced cardiomyopathy. E_rr_ estimation has historically been a more difficult metric to measure consistently. For example, Cao et al. showed moderate differences in E_cc_ and E_ll_ between vendors using CMR FT, but very large differences in E_rr_ [57]. Despite these considerations, we observed that E_rr_ may still be a valuable metric and warrants further validation and comparison in DMD populations.

We also explored the use of E_a_ and E_a_ strain rate metrics from 3D + time CMR imaging in DMD patients. This metric, while unique to CMR analysis in DMD patients, has been explored with 3D speckle tracking echocardiography (3D-STE) in DMD CMP. For example, Yu et al*.* demonstrated that E_a_ derived from 3D-STE had an 85.7% sensitivity and a 71.0% specificity for differentiating DMD patients (n = 56) from controls (n = 31) [58]. E_a_ is a relatively novel metric, unique to 3D imaging, that takes into account both longitudinal and circumferential shortening [59]. Also, since the myocardium is relatively incompressible, radial thickening during systole influences E_a_ setting up an inverse relationship between E_a_ and E_rr_. The integration of these effects into a single strain parameter makes E_a_ potentially useful in examining subclinical dysfunction. Since this is a relatively novel parameter, more studies are needed to determine its full value, especially in CMR imaging. Conventional echocardiographic strain estimation techniques often have a higher temporal resolution (< 5 ms) compared to CMR (20–50 ms), though CMR offers superior contrast resolution [50]. Additionally, methodological differences make a direct comparison of strain values between STE and CMR difficult; depending on technique and study population, these values may not be in agreement [60]. Transthoracic echocardiography (TTE) is used for the initial screening of cardiac function in nearly every patient population, including those at risk for DMD. However, as the disease progresses, limited TTE windows and image artifacts due to scoliosis and fat deposition make cardiac assessment with TTE increasingly difficult [61]. In many DMD patients, only a small number of measures of LV function can be reliably estimated from TTE [61]. Thus, while 3D-STE is a promising characterization technique in some patients, CMR remains the gold standard for evaluation in this patient population.

Many other strain imaging techniques are currently being used to analyze CMR data. The technique described in this work is best characterized as a 3D feature-tracking Lagrangian deformation estimation technique that utilizes image features in CMR scans to estimate strain. Ventricular boundaries, brightness, and homogeneity are all tracked throughout the cardiac cycle to produce deformation. Others have used similar 2D feature-tracking techniques in DMD cohorts to detect morphologic changes in the absence of LGE as well as to detect changes between DMD and healthy subjects not seen using 3D-STE [62]. One major benefit to the feature-tracking approach we use is that it utilizes conventional short-axis cine images and thus does not increase the complexity or length of a typical CMR imaging protocol. Another major advantage of our platform is that it provides additional and more comprehensive 3D imaging strain metrics (i.e. surface area strain, strain rate) compared to conventional metrics typically available through other commercial platforms. The nature of our platform also allows for raw export of image and segmentation data for further, more extensive analysis- one example being the 48-point surface representation of the ventricle which lends itself well to machine and deep learning algorithms. We note as well that this 48-point surface representation does not employ a more traditional basal, mid-ventricular, and apical sections, but rather utilizes a 4-slice length-based analysis (25%, 50%, 75%, 100%). We found that the use of this 4-slice representation allowed for reasonably accurate feature tracking while also permitting a simple and rapid analysis. One drawback to this method is the need to interpolate between slices. A benefit to this approach, however, is that the segmentation framework is adaptable such that more or less slices can easily be added to enhance future analysis. A major drawback to our method is that the post-processing steps for each scan require a moderate amount of training and additional time to complete. Non-expert, non-clinical users of the graphical user interface felt comfortable with its use after 20–30 min of training. After some practice, users were able to complete a full 4D analysis in 30–45 min. While this analysis time is likely not feasible outside of a research setting, efforts are being made to further simplify this approach and incorporate machine learning techniques to automate manual segmentation and analysis [24].

It is important to note that all CMR strain imaging techniques have benefits and drawbacks. HARmonic Phase (HARP) analysis is perhaps the most utilized strain imaging method incorporated into the largest number of post-processing software packages [63–65]. HARP is a CMR tagging strain analysis technique that isolates one Fourier component of the amplitude modulated data, and tracks pixels with consistent phase [64]. This allows for a relatively rapid and reproducible strain estimation compared to other techniques that involve additional scan time. HARP analysis in conjunction with CMR tagging has been used to reliably estimate strain in DMD patients allowing for robust patient stratification [46, 47]. While this technique is useful in many clinical scenarios, additional scan time, lack of standardization, low spatial resolution, and tag fading are all drawbacks to this method [66, 67].

Displacement encoding with stimulated echoes (DENSE), strain encoded CMR (SENC), and tissue phase mapping (TPM) are additional methods for strain estimation using CMR, but these are less studied in DMD populations [46, 47]. DENSE is generally accepted as an accurate and reproducible method for strain imaging as it relies on the phase information of a stimulated echo and is directly proportional to tissue displacement. By analyzing directional-encoded phase images, the Lagrangian displacement fields can be produced. SENC is similar to HARP in that it utilizes parallel tagged lines with out-of-plane phase encoding gradients to estimate strain. Finally, TPM relies solely on the pixel phase from which it encodes velocity from each image allowing for spatial integration and estimation of deformation and strain. Each of these techniques relies on specialized image sequences and takes additional scan time, but they produce relatively high spatial and temporal resolution needed for strain estimates. In addition, each of these methods is not widely used clinically, though they are being used in research studies [68–70]. Each of the additional CMR strain imaging techniques mentioned (HARP, DENSE, SENC, and TPM) are most often used on 2D images, though they could be used in 3D reconstruction techniques for strain estimation. Fully 3D CMR acquisition is being explored but the acquisition time is longer than conventional 2D scanning. Additionally, techniques must be robust enough to overcome motion artifacts, and excitation of 3D volume may diminish image contrast between blood and myocardium [71].

The primary purpose and scope of this work was to determine the feasibility and utility of our 3D cine strain analysis technique in DMD CMP. While we see similar differences between DMD and healthy control subjects, the ranges for strain values may differ slightly from other techniques. This is not a unique issue in our proposed method as studies have shown differences in 2D CMR strain where FT techniques and in different cardiac pathologies. For example, Chew et al. demonstrated that CMR FT may overestimate strain when compared to SENC in adult and pediatric congenital heart disease cohorts [72]. Others have shown good agreement between CMR FT and HARP measuring E_cc_ in a DMD population [73], and CMR FT and DENSE in adults with myocardial infarction [74]. Even when using CMR FT on the same cohort of patients with CMP, inter-vendor differences were found in E_cc_ and E_ll_ and E_rr_ [57]. Small differences have also been shown between 2 and 3D derived CMR FT strain values in DMD [48] and healthy adult populations [49]. As with most sequential cine acquisitions it is also possible that we are very slightly underestimating peak strain because our raw image temporal resolution (20–25 images per cardiac cycle) may not perfectly capture the moment of peak myocardial contraction especially with higher heartrate. Thus, as with any other technique, one should interpret the control and DMD strain value ranges considering these differences between techniques, vendors, and disease characteristics. Future prospective studies will be needed to fully compare and validate the approach described here with 2D and 3D CMR strain imaging data acquired from the same patients. Analysis of a larger longitudinal cohort will also allow us to determine if we can predict outcomes from the novel metrics we derive from our 4D CMR strain method.

### Limitations

Our study has several limitations. Although we noted significant differences in many strain metrics between DMD and healthy control subjects, this is a cross-sectional study and not a longitudinal study. As such, longitudinal analysis of patients would add stronger evidence as to whether these measures are sensitive and specific and can determine longitudinal changes that may correlate with disease progression, and even mortality. For this study we considered 43 DMD CMP patients and 25 healthy control subjects. While we were able to perform analysis on an age- and sex-matched cohort, this relatively smaller sample size did not allow for robust modeling to account for all confounding factors such as height, weight, or blood pressures. A larger study may be needed to fully validate these findings in light of many potential confounders. Additionally, for many of our strain metrics (E_ll_, E_rr_, and E_a_) we used the small strain linear approximation which may not be as accurate as a finite strain approximation for larger strain values. That said, the relative differences between DMD and healthy subjects should still be evident. Another limitation in data analysis is the labor-intensive process to contour and segment the LV while considering CMR image artifacts caused by gross movement of the patient or diaphragm necessitating manual correction. An automated or even semi-automated approach using machine learning techniques to correct displacement and aid in segmentation may help this process [24]. Further, the short axis cine images used to create the 3D + time datasets are acquired sequentially and not simultaneously. In addition, the use of a simplified 48-point representation to create a 3D dynamic mesh relies on both spatial and temporal interpolation. While this representation may lend itself well to a simplified feature-tracking method, further work will be needed to validate its use.

## Conclusion

In this study, we demonstrate a novel 3D + time CMR imaging analysis platform and describe sensitive and specific strain and strain rate metrics in DMD patients that differentiate mild cardiac disease from healthy subjects and correlate with LVEF and ECV. We also describe for the first time Ea and strain rate metrics derived from 3D cine CMR imaging in a cohort of DMD patients that significantly differentiate these patients from healthy subjects. Identification of sensitive imaging markers for CMP onset and progression could guide prospective therapeutic intervention and refine the power for clinical trials aimed to improve outcomes and therapies in this vulnerable population.

## Supplementary Information

**Additional file 1. **Circumferential (E_cc_) colorized strain map derived from 3D+time CMR images of a DMD patient from MATLAB feature-tracking analysis.

**Additional file 2. **Longitudinal (E_ll_) colorized strain map derived from 3D+time CMR images of a DMD patient from MATLAB feature-tracking analysis.

**Additional file 3. **Radial (E_rr_) colorized strain map derived from 3D+time CMR images of a DMD patient from MATLAB feature-tracking analysis.

**Additional file 4. **Surface area (E_a_) colorized strain map derived from 3D+time CMR images of a DMD patient from MATLAB feature-tracking analysis.

**Additional file 5. **Regional peak strain, systolic, strain rate, early diastolic strain rate, and late diastolic strain rate derived from 3D+time CMR images are significantly different between DMD CMP (n=43) and healthy control subjects (n=25) and strongly discriminate between disease and healthy controls based on AUC analysis.

**Additional file 6. **17 segment characterization of radial and surface area regional peak strain, systolic early diastolic, and late diastolic strain rate derived from 3D+time CMR images significantly different between DMD CM (n=43) and healthy control subjects (n=25) and strongly discriminate between disease and healthy controls based on AUC analysis.

**Additional file 7. **Late gadolinium enhancement (LGE) distribution in DMD CMP patients. A) 17-segment bullseye map depicting regions of the LV B) Control (n=12) and C) DMD-associated cardiomyopathy (DMD CMP; n=43) colorized bullseye maps showing percentage of cohort with LGE in a particular segment of the LV. D) Bar graph showing percentage of DMD CMP cohort with LGE in each of the 17 segments.

**Additional file 8. **Regional strain correlation (Pearson’s r) for peak strain and systolic strain rate. S- septal, FW- free-wall.

**Additional file 9. **Peak and systolic strain rate regional correlation (Pearson’s r). significant AFW- Anterior Free Wall, A- Anterior, AS- Anterior Septal, PS- Posterior Septal, P- Posterior, PFW- Posterior Free Wall, P-Peak, SR-Strain Rate.

## Data Availability

The datasets and analysis tools used during this study are available from the corresponding author on reasonable request.

## Abbreviations

AHA: American Heart Association
AUC: Area under the curve
bSSFP: Balanced steady state free precession
CMP: Cardiomyopathy
CMR: Cardiovascular magnetic resonance
CO: Cardiac output
DENSE: Displacement encoding with stimulated echos
DMD: Duchenne muscular dystrophy
Ea: Surface area strain
E_cc_: Circumferential strain
E_ll_: Longitudinal strain
E_rr_: Radial strain
ECG: Electrocardiogram
ECV: Extracellular volume
FT: Feature tracking
HARP: Harmonic phase
LGE: Late gadolinium enhancement
LV: Left ventricle/left ventricular
LVEDVI: Left ventricular end diastolic volume indexed to body surface area
LVEF: Left ventricular ejection fraction
LVESVI: Left ventricular end systolic volume indexed to body surface area
MOLLI: Modified Look-Locker inversion recovery
MDE: Myocardial delayed enhancement
PSIR: Phase sensitive inversion recovery
ROC: Receiver operator curve
SENC: Strain encoding
STE: Speckle tracking echocardiography
TPM: Tissue phase mapping
TTE: Transthoracic echocardiography
