# Supplementary material for: Localized strain characterization of cardiomyopathy in Duchenne muscular dystrophy using novel 4D kinematic analysis of cine cardiovascular magnetic resonance
Source: J Cardiovasc Magn Reson. 2023 Feb 16;25:14. doi: 10.1186/s12968-023-00922-3 (PMC9933368; doi:10.1186/s12968-023-00922-3)
Supplement: Supplementary file 5 — Additional file 5. Regional peak strain, systolic, strain rate, early diastolic strain rate, and late diastolic strain rate derived from 3D+time CMR images are significantly different between DMD CMP (n=43) and healthy control subjects (n=25) and strongly discriminate between disease and healthy controls based on AUC analysis. [file 12968_2023_922_MOESM5_ESM.pptx]

## Slide 1
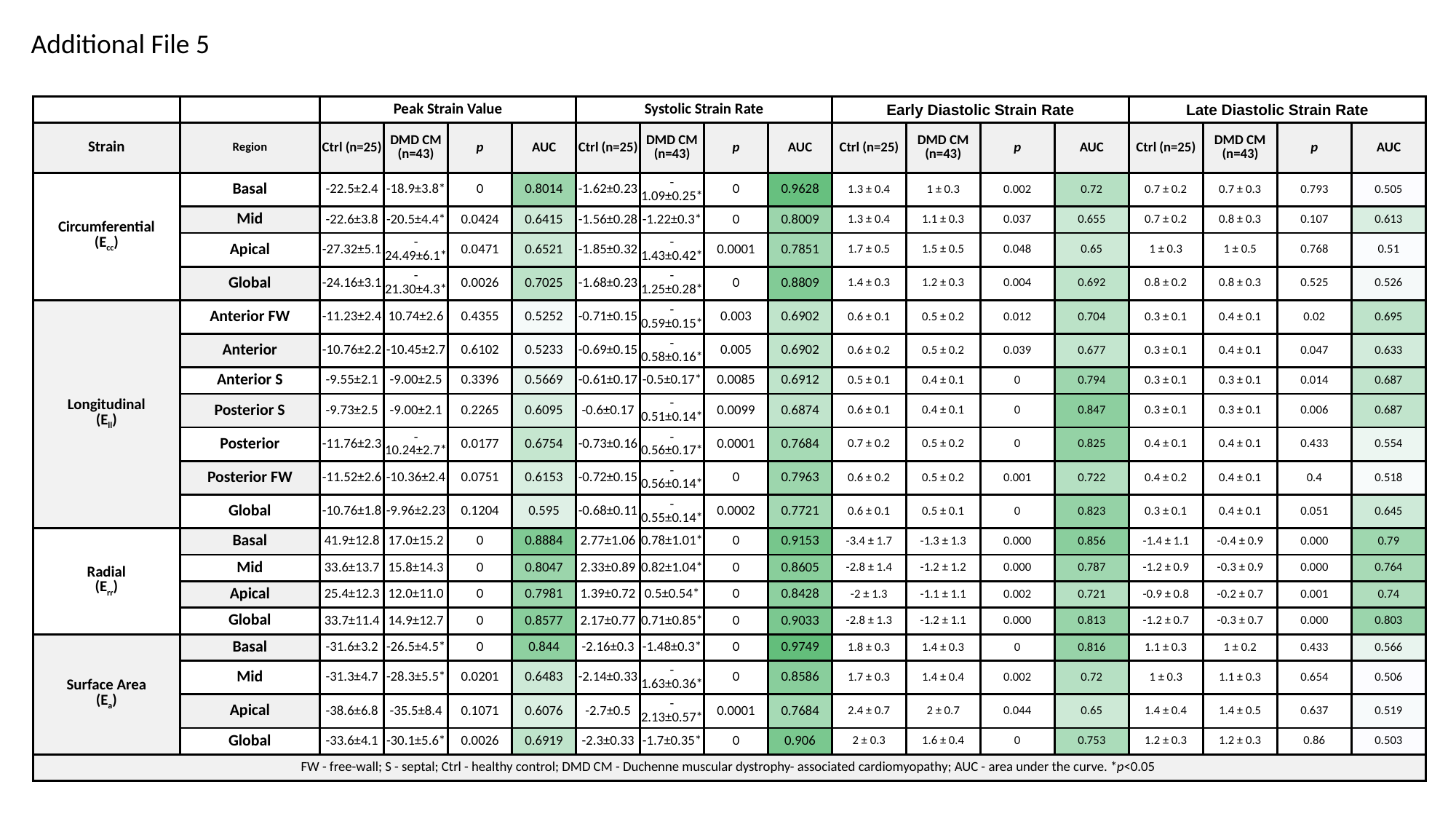

Additional File 5
| | | Peak Strain Value | | | | Systolic Strain Rate | | | | Early Diastolic Strain Rate | | | | Late Diastolic Strain Rate | | | |
| --- | --- | --- | --- | --- | --- | --- | --- | --- | --- | --- | --- | --- | --- | --- | --- | --- | --- |
| Strain | Region | Ctrl (n=25) | DMD CM (n=43) | p | AUC | Ctrl (n=25) | DMD CM (n=43) | p | AUC | Ctrl (n=25) | DMD CM (n=43) | p | AUC | Ctrl (n=25) | DMD CM (n=43) | p | AUC |
| Circumferential (Ecc) | Basal | -22.5±2.4 | -18.9±3.8\* | 0 | 0.8014 | -1.62±0.23 | -1.09±0.25\* | 0 | 0.9628 | 1.3 ± 0.4 | 1 ± 0.3 | 0.002 | 0.72 | 0.7 ± 0.2 | 0.7 ± 0.3 | 0.793 | 0.505 |
| | Mid | -22.6±3.8 | -20.5±4.4\* | 0.0424 | 0.6415 | -1.56±0.28 | -1.22±0.3\* | 0 | 0.8009 | 1.3 ± 0.4 | 1.1 ± 0.3 | 0.037 | 0.655 | 0.7 ± 0.2 | 0.8 ± 0.3 | 0.107 | 0.613 |
| | Apical | -27.32±5.1 | -24.49±6.1\* | 0.0471 | 0.6521 | -1.85±0.32 | -1.43±0.42\* | 0.0001 | 0.7851 | 1.7 ± 0.5 | 1.5 ± 0.5 | 0.048 | 0.65 | 1 ± 0.3 | 1 ± 0.5 | 0.768 | 0.51 |
| | Global | -24.16±3.1 | -21.30±4.3\* | 0.0026 | 0.7025 | -1.68±0.23 | -1.25±0.28\* | 0 | 0.8809 | 1.4 ± 0.3 | 1.2 ± 0.3 | 0.004 | 0.692 | 0.8 ± 0.2 | 0.8 ± 0.3 | 0.525 | 0.526 |
| Longitudinal (Ell) | Anterior FW | -11.23±2.4 | 10.74±2.6 | 0.4355 | 0.5252 | -0.71±0.15 | -0.59±0.15\* | 0.003 | 0.6902 | 0.6 ± 0.1 | 0.5 ± 0.2 | 0.012 | 0.704 | 0.3 ± 0.1 | 0.4 ± 0.1 | 0.02 | 0.695 |
| | Anterior | -10.76±2.2 | -10.45±2.7 | 0.6102 | 0.5233 | -0.69±0.15 | -0.58±0.16\* | 0.005 | 0.6902 | 0.6 ± 0.2 | 0.5 ± 0.2 | 0.039 | 0.677 | 0.3 ± 0.1 | 0.4 ± 0.1 | 0.047 | 0.633 |
| | Anterior S | -9.55±2.1 | -9.00±2.5 | 0.3396 | 0.5669 | -0.61±0.17 | -0.5±0.17\* | 0.0085 | 0.6912 | 0.5 ± 0.1 | 0.4 ± 0.1 | 0 | 0.794 | 0.3 ± 0.1 | 0.3 ± 0.1 | 0.014 | 0.687 |
| | Posterior S | -9.73±2.5 | -9.00±2.1 | 0.2265 | 0.6095 | -0.6±0.17 | -0.51±0.14\* | 0.0099 | 0.6874 | 0.6 ± 0.1 | 0.4 ± 0.1 | 0 | 0.847 | 0.3 ± 0.1 | 0.3 ± 0.1 | 0.006 | 0.687 |
| | Posterior | -11.76±2.3 | -10.24±2.7\* | 0.0177 | 0.6754 | -0.73±0.16 | -0.56±0.17\* | 0.0001 | 0.7684 | 0.7 ± 0.2 | 0.5 ± 0.2 | 0 | 0.825 | 0.4 ± 0.1 | 0.4 ± 0.1 | 0.433 | 0.554 |
| | Posterior FW | -11.52±2.6 | -10.36±2.4 | 0.0751 | 0.6153 | -0.72±0.15 | -0.56±0.14\* | 0 | 0.7963 | 0.6 ± 0.2 | 0.5 ± 0.2 | 0.001 | 0.722 | 0.4 ± 0.2 | 0.4 ± 0.1 | 0.4 | 0.518 |
| | Global | -10.76±1.8 | -9.96±2.23 | 0.1204 | 0.595 | -0.68±0.11 | -0.55±0.14\* | 0.0002 | 0.7721 | 0.6 ± 0.1 | 0.5 ± 0.1 | 0 | 0.823 | 0.3 ± 0.1 | 0.4 ± 0.1 | 0.051 | 0.645 |
| Radial (Err) | Basal | 41.9±12.8 | 17.0±15.2 | 0 | 0.8884 | 2.77±1.06 | 0.78±1.01\* | 0 | 0.9153 | -3.4 ± 1.7 | -1.3 ± 1.3 | 0.000 | 0.856 | -1.4 ± 1.1 | -0.4 ± 0.9 | 0.000 | 0.79 |
| | Mid | 33.6±13.7 | 15.8±14.3 | 0 | 0.8047 | 2.33±0.89 | 0.82±1.04\* | 0 | 0.8605 | -2.8 ± 1.4 | -1.2 ± 1.2 | 0.000 | 0.787 | -1.2 ± 0.9 | -0.3 ± 0.9 | 0.000 | 0.764 |
| | Apical | 25.4±12.3 | 12.0±11.0 | 0 | 0.7981 | 1.39±0.72 | 0.5±0.54\* | 0 | 0.8428 | -2 ± 1.3 | -1.1 ± 1.1 | 0.002 | 0.721 | -0.9 ± 0.8 | -0.2 ± 0.7 | 0.001 | 0.74 |
| | Global | 33.7±11.4 | 14.9±12.7 | 0 | 0.8577 | 2.17±0.77 | 0.71±0.85\* | 0 | 0.9033 | -2.8 ± 1.3 | -1.2 ± 1.1 | 0.000 | 0.813 | -1.2 ± 0.7 | -0.3 ± 0.7 | 0.000 | 0.803 |
| Surface Area (Ea) | Basal | -31.6±3.2 | -26.5±4.5\* | 0 | 0.844 | -2.16±0.3 | -1.48±0.3\* | 0 | 0.9749 | 1.8 ± 0.3 | 1.4 ± 0.3 | 0 | 0.816 | 1.1 ± 0.3 | 1 ± 0.2 | 0.433 | 0.566 |
| | Mid | -31.3±4.7 | -28.3±5.5\* | 0.0201 | 0.6483 | -2.14±0.33 | -1.63±0.36\* | 0 | 0.8586 | 1.7 ± 0.3 | 1.4 ± 0.4 | 0.002 | 0.72 | 1 ± 0.3 | 1.1 ± 0.3 | 0.654 | 0.506 |
| | Apical | -38.6±6.8 | -35.5±8.4 | 0.1071 | 0.6076 | -2.7±0.5 | -2.13±0.57\* | 0.0001 | 0.7684 | 2.4 ± 0.7 | 2 ± 0.7 | 0.044 | 0.65 | 1.4 ± 0.4 | 1.4 ± 0.5 | 0.637 | 0.519 |
| | Global | -33.6±4.1 | -30.1±5.6\* | 0.0026 | 0.6919 | -2.3±0.33 | -1.7±0.35\* | 0 | 0.906 | 2 ± 0.3 | 1.6 ± 0.4 | 0 | 0.753 | 1.2 ± 0.3 | 1.2 ± 0.3 | 0.86 | 0.503 |
| FW - free-wall; S - septal; Ctrl - healthy control; DMD CM - Duchenne muscular dystrophy- associated cardiomyopathy; AUC - area under the curve. \*p<0.05 | | | | | | | | | | | | | | | | | |
