# Supplementary material for: Localized strain characterization of cardiomyopathy in Duchenne muscular dystrophy using novel 4D kinematic analysis of cine cardiovascular magnetic resonance
Source: J Cardiovasc Magn Reson. 2023 Feb 16;25:14. doi: 10.1186/s12968-023-00922-3 (PMC9933368; doi:10.1186/s12968-023-00922-3)
Supplement: Supplementary file 6 — Additional file 6. 17 segment characterization of radial and surface area regional peak strain, systolic early diastolic, and late diastolic strain rate derived from 3D+time CMR images significantly different between DMD CM (n=43) and healthy control subjects (n=25) and strongly discriminate between disease and healthy controls based on AUC analysis. [file 12968_2023_922_MOESM6_ESM.pptx]

## Slide 1
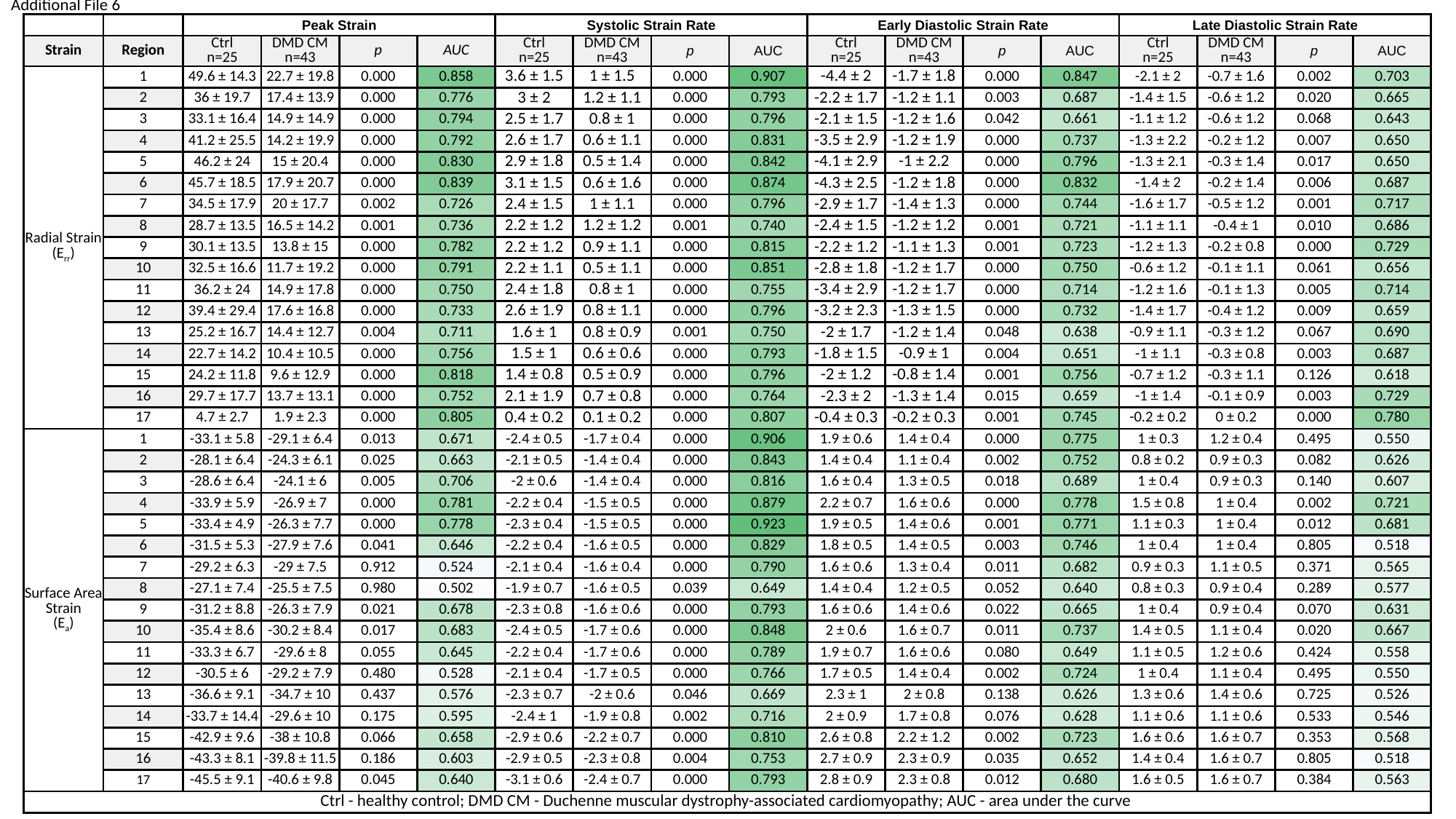

Additional File 6
| | | Peak Strain | | | | Systolic Strain Rate | | | | Early Diastolic Strain Rate | | | | Late Diastolic Strain Rate | | | |
| --- | --- | --- | --- | --- | --- | --- | --- | --- | --- | --- | --- | --- | --- | --- | --- | --- | --- |
| Strain | Region | Ctrl n=25 | DMD CM n=43 | p | AUC | Ctrl n=25 | DMD CM n=43 | p | AUC | Ctrl n=25 | DMD CM n=43 | p | AUC | Ctrl n=25 | DMD CM n=43 | p | AUC |
| Radial Strain (Err) | 1 | 49.6 ± 14.3 | 22.7 ± 19.8 | 0.000 | 0.858 | 3.6 ± 1.5 | 1 ± 1.5 | 0.000 | 0.907 | -4.4 ± 2 | -1.7 ± 1.8 | 0.000 | 0.847 | -2.1 ± 2 | -0.7 ± 1.6 | 0.002 | 0.703 |
| | 2 | 36 ± 19.7 | 17.4 ± 13.9 | 0.000 | 0.776 | 3 ± 2 | 1.2 ± 1.1 | 0.000 | 0.793 | -2.2 ± 1.7 | -1.2 ± 1.1 | 0.003 | 0.687 | -1.4 ± 1.5 | -0.6 ± 1.2 | 0.020 | 0.665 |
| | 3 | 33.1 ± 16.4 | 14.9 ± 14.9 | 0.000 | 0.794 | 2.5 ± 1.7 | 0.8 ± 1 | 0.000 | 0.796 | -2.1 ± 1.5 | -1.2 ± 1.6 | 0.042 | 0.661 | -1.1 ± 1.2 | -0.6 ± 1.2 | 0.068 | 0.643 |
| | 4 | 41.2 ± 25.5 | 14.2 ± 19.9 | 0.000 | 0.792 | 2.6 ± 1.7 | 0.6 ± 1.1 | 0.000 | 0.831 | -3.5 ± 2.9 | -1.2 ± 1.9 | 0.000 | 0.737 | -1.3 ± 2.2 | -0.2 ± 1.2 | 0.007 | 0.650 |
| | 5 | 46.2 ± 24 | 15 ± 20.4 | 0.000 | 0.830 | 2.9 ± 1.8 | 0.5 ± 1.4 | 0.000 | 0.842 | -4.1 ± 2.9 | -1 ± 2.2 | 0.000 | 0.796 | -1.3 ± 2.1 | -0.3 ± 1.4 | 0.017 | 0.650 |
| | 6 | 45.7 ± 18.5 | 17.9 ± 20.7 | 0.000 | 0.839 | 3.1 ± 1.5 | 0.6 ± 1.6 | 0.000 | 0.874 | -4.3 ± 2.5 | -1.2 ± 1.8 | 0.000 | 0.832 | -1.4 ± 2 | -0.2 ± 1.4 | 0.006 | 0.687 |
| | 7 | 34.5 ± 17.9 | 20 ± 17.7 | 0.002 | 0.726 | 2.4 ± 1.5 | 1 ± 1.1 | 0.000 | 0.796 | -2.9 ± 1.7 | -1.4 ± 1.3 | 0.000 | 0.744 | -1.6 ± 1.7 | -0.5 ± 1.2 | 0.001 | 0.717 |
| | 8 | 28.7 ± 13.5 | 16.5 ± 14.2 | 0.001 | 0.736 | 2.2 ± 1.2 | 1.2 ± 1.2 | 0.001 | 0.740 | -2.4 ± 1.5 | -1.2 ± 1.2 | 0.001 | 0.721 | -1.1 ± 1.1 | -0.4 ± 1 | 0.010 | 0.686 |
| | 9 | 30.1 ± 13.5 | 13.8 ± 15 | 0.000 | 0.782 | 2.2 ± 1.2 | 0.9 ± 1.1 | 0.000 | 0.815 | -2.2 ± 1.2 | -1.1 ± 1.3 | 0.001 | 0.723 | -1.2 ± 1.3 | -0.2 ± 0.8 | 0.000 | 0.729 |
| | 10 | 32.5 ± 16.6 | 11.7 ± 19.2 | 0.000 | 0.791 | 2.2 ± 1.1 | 0.5 ± 1.1 | 0.000 | 0.851 | -2.8 ± 1.8 | -1.2 ± 1.7 | 0.000 | 0.750 | -0.6 ± 1.2 | -0.1 ± 1.1 | 0.061 | 0.656 |
| | 11 | 36.2 ± 24 | 14.9 ± 17.8 | 0.000 | 0.750 | 2.4 ± 1.8 | 0.8 ± 1 | 0.000 | 0.755 | -3.4 ± 2.9 | -1.2 ± 1.7 | 0.000 | 0.714 | -1.2 ± 1.6 | -0.1 ± 1.3 | 0.005 | 0.714 |
| | 12 | 39.4 ± 29.4 | 17.6 ± 16.8 | 0.000 | 0.733 | 2.6 ± 1.9 | 0.8 ± 1.1 | 0.000 | 0.796 | -3.2 ± 2.3 | -1.3 ± 1.5 | 0.000 | 0.732 | -1.4 ± 1.7 | -0.4 ± 1.2 | 0.009 | 0.659 |
| | 13 | 25.2 ± 16.7 | 14.4 ± 12.7 | 0.004 | 0.711 | 1.6 ± 1 | 0.8 ± 0.9 | 0.001 | 0.750 | -2 ± 1.7 | -1.2 ± 1.4 | 0.048 | 0.638 | -0.9 ± 1.1 | -0.3 ± 1.2 | 0.067 | 0.690 |
| | 14 | 22.7 ± 14.2 | 10.4 ± 10.5 | 0.000 | 0.756 | 1.5 ± 1 | 0.6 ± 0.6 | 0.000 | 0.793 | -1.8 ± 1.5 | -0.9 ± 1 | 0.004 | 0.651 | -1 ± 1.1 | -0.3 ± 0.8 | 0.003 | 0.687 |
| | 15 | 24.2 ± 11.8 | 9.6 ± 12.9 | 0.000 | 0.818 | 1.4 ± 0.8 | 0.5 ± 0.9 | 0.000 | 0.796 | -2 ± 1.2 | -0.8 ± 1.4 | 0.001 | 0.756 | -0.7 ± 1.2 | -0.3 ± 1.1 | 0.126 | 0.618 |
| | 16 | 29.7 ± 17.7 | 13.7 ± 13.1 | 0.000 | 0.752 | 2.1 ± 1.9 | 0.7 ± 0.8 | 0.000 | 0.764 | -2.3 ± 2 | -1.3 ± 1.4 | 0.015 | 0.659 | -1 ± 1.4 | -0.1 ± 0.9 | 0.003 | 0.729 |
| | 17 | 4.7 ± 2.7 | 1.9 ± 2.3 | 0.000 | 0.805 | 0.4 ± 0.2 | 0.1 ± 0.2 | 0.000 | 0.807 | -0.4 ± 0.3 | -0.2 ± 0.3 | 0.001 | 0.745 | -0.2 ± 0.2 | 0 ± 0.2 | 0.000 | 0.780 |
| Surface Area Strain (Ea) | 1 | -33.1 ± 5.8 | -29.1 ± 6.4 | 0.013 | 0.671 | -2.4 ± 0.5 | -1.7 ± 0.4 | 0.000 | 0.906 | 1.9 ± 0.6 | 1.4 ± 0.4 | 0.000 | 0.775 | 1 ± 0.3 | 1.2 ± 0.4 | 0.495 | 0.550 |
| | 2 | -28.1 ± 6.4 | -24.3 ± 6.1 | 0.025 | 0.663 | -2.1 ± 0.5 | -1.4 ± 0.4 | 0.000 | 0.843 | 1.4 ± 0.4 | 1.1 ± 0.4 | 0.002 | 0.752 | 0.8 ± 0.2 | 0.9 ± 0.3 | 0.082 | 0.626 |
| | 3 | -28.6 ± 6.4 | -24.1 ± 6 | 0.005 | 0.706 | -2 ± 0.6 | -1.4 ± 0.4 | 0.000 | 0.816 | 1.6 ± 0.4 | 1.3 ± 0.5 | 0.018 | 0.689 | 1 ± 0.4 | 0.9 ± 0.3 | 0.140 | 0.607 |
| | 4 | -33.9 ± 5.9 | -26.9 ± 7 | 0.000 | 0.781 | -2.2 ± 0.4 | -1.5 ± 0.5 | 0.000 | 0.879 | 2.2 ± 0.7 | 1.6 ± 0.6 | 0.000 | 0.778 | 1.5 ± 0.8 | 1 ± 0.4 | 0.002 | 0.721 |
| | 5 | -33.4 ± 4.9 | -26.3 ± 7.7 | 0.000 | 0.778 | -2.3 ± 0.4 | -1.5 ± 0.5 | 0.000 | 0.923 | 1.9 ± 0.5 | 1.4 ± 0.6 | 0.001 | 0.771 | 1.1 ± 0.3 | 1 ± 0.4 | 0.012 | 0.681 |
| | 6 | -31.5 ± 5.3 | -27.9 ± 7.6 | 0.041 | 0.646 | -2.2 ± 0.4 | -1.6 ± 0.5 | 0.000 | 0.829 | 1.8 ± 0.5 | 1.4 ± 0.5 | 0.003 | 0.746 | 1 ± 0.4 | 1 ± 0.4 | 0.805 | 0.518 |
| | 7 | -29.2 ± 6.3 | -29 ± 7.5 | 0.912 | 0.524 | -2.1 ± 0.4 | -1.6 ± 0.4 | 0.000 | 0.790 | 1.6 ± 0.6 | 1.3 ± 0.4 | 0.011 | 0.682 | 0.9 ± 0.3 | 1.1 ± 0.5 | 0.371 | 0.565 |
| | 8 | -27.1 ± 7.4 | -25.5 ± 7.5 | 0.980 | 0.502 | -1.9 ± 0.7 | -1.6 ± 0.5 | 0.039 | 0.649 | 1.4 ± 0.4 | 1.2 ± 0.5 | 0.052 | 0.640 | 0.8 ± 0.3 | 0.9 ± 0.4 | 0.289 | 0.577 |
| | 9 | -31.2 ± 8.8 | -26.3 ± 7.9 | 0.021 | 0.678 | -2.3 ± 0.8 | -1.6 ± 0.6 | 0.000 | 0.793 | 1.6 ± 0.6 | 1.4 ± 0.6 | 0.022 | 0.665 | 1 ± 0.4 | 0.9 ± 0.4 | 0.070 | 0.631 |
| | 10 | -35.4 ± 8.6 | -30.2 ± 8.4 | 0.017 | 0.683 | -2.4 ± 0.5 | -1.7 ± 0.6 | 0.000 | 0.848 | 2 ± 0.6 | 1.6 ± 0.7 | 0.011 | 0.737 | 1.4 ± 0.5 | 1.1 ± 0.4 | 0.020 | 0.667 |
| | 11 | -33.3 ± 6.7 | -29.6 ± 8 | 0.055 | 0.645 | -2.2 ± 0.4 | -1.7 ± 0.6 | 0.000 | 0.789 | 1.9 ± 0.7 | 1.6 ± 0.6 | 0.080 | 0.649 | 1.1 ± 0.5 | 1.2 ± 0.6 | 0.424 | 0.558 |
| | 12 | -30.5 ± 6 | -29.2 ± 7.9 | 0.480 | 0.528 | -2.1 ± 0.4 | -1.7 ± 0.5 | 0.000 | 0.766 | 1.7 ± 0.5 | 1.4 ± 0.4 | 0.002 | 0.724 | 1 ± 0.4 | 1.1 ± 0.4 | 0.495 | 0.550 |
| | 13 | -36.6 ± 9.1 | -34.7 ± 10 | 0.437 | 0.576 | -2.3 ± 0.7 | -2 ± 0.6 | 0.046 | 0.669 | 2.3 ± 1 | 2 ± 0.8 | 0.138 | 0.626 | 1.3 ± 0.6 | 1.4 ± 0.6 | 0.725 | 0.526 |
| | 14 | -33.7 ± 14.4 | -29.6 ± 10 | 0.175 | 0.595 | -2.4 ± 1 | -1.9 ± 0.8 | 0.002 | 0.716 | 2 ± 0.9 | 1.7 ± 0.8 | 0.076 | 0.628 | 1.1 ± 0.6 | 1.1 ± 0.6 | 0.533 | 0.546 |
| | 15 | -42.9 ± 9.6 | -38 ± 10.8 | 0.066 | 0.658 | -2.9 ± 0.6 | -2.2 ± 0.7 | 0.000 | 0.810 | 2.6 ± 0.8 | 2.2 ± 1.2 | 0.002 | 0.723 | 1.6 ± 0.6 | 1.6 ± 0.7 | 0.353 | 0.568 |
| | 16 | -43.3 ± 8.1 | -39.8 ± 11.5 | 0.186 | 0.603 | -2.9 ± 0.5 | -2.3 ± 0.8 | 0.004 | 0.753 | 2.7 ± 0.9 | 2.3 ± 0.9 | 0.035 | 0.652 | 1.4 ± 0.4 | 1.6 ± 0.7 | 0.805 | 0.518 |
| | 17 | -45.5 ± 9.1 | -40.6 ± 9.8 | 0.045 | 0.640 | -3.1 ± 0.6 | -2.4 ± 0.7 | 0.000 | 0.793 | 2.8 ± 0.9 | 2.3 ± 0.8 | 0.012 | 0.680 | 1.6 ± 0.5 | 1.6 ± 0.7 | 0.384 | 0.563 |
| Ctrl - healthy control; DMD CM - Duchenne muscular dystrophy-associated cardiomyopathy; AUC - area under the curve | | | | | | | | | | | | | | | | | |
