# Supplementary material for: Localized strain characterization of cardiomyopathy in Duchenne muscular dystrophy using novel 4D kinematic analysis of cine cardiovascular magnetic resonance
Source: J Cardiovasc Magn Reson. 2023 Feb 16;25:14. doi: 10.1186/s12968-023-00922-3 (PMC9933368; doi:10.1186/s12968-023-00922-3)
Supplement: Supplementary file 7 — Additional file 7. Late gadolinium enhancement (LGE) distribution in DMD CMP patients. A) 17-segment bullseye map depicting regions of the LV B) Control (n=12) and C) DMD-associated cardiomyopathy (DMD CMP; n=43) colorized bullseye maps showing percentage of cohort with LGE in a particular segment of the LV. D) Bar graph showing percentage of DMD CMP cohort with LGE in each of the 17 segments. [file 12968_2023_922_MOESM7_ESM.pptx]

## Slide 1
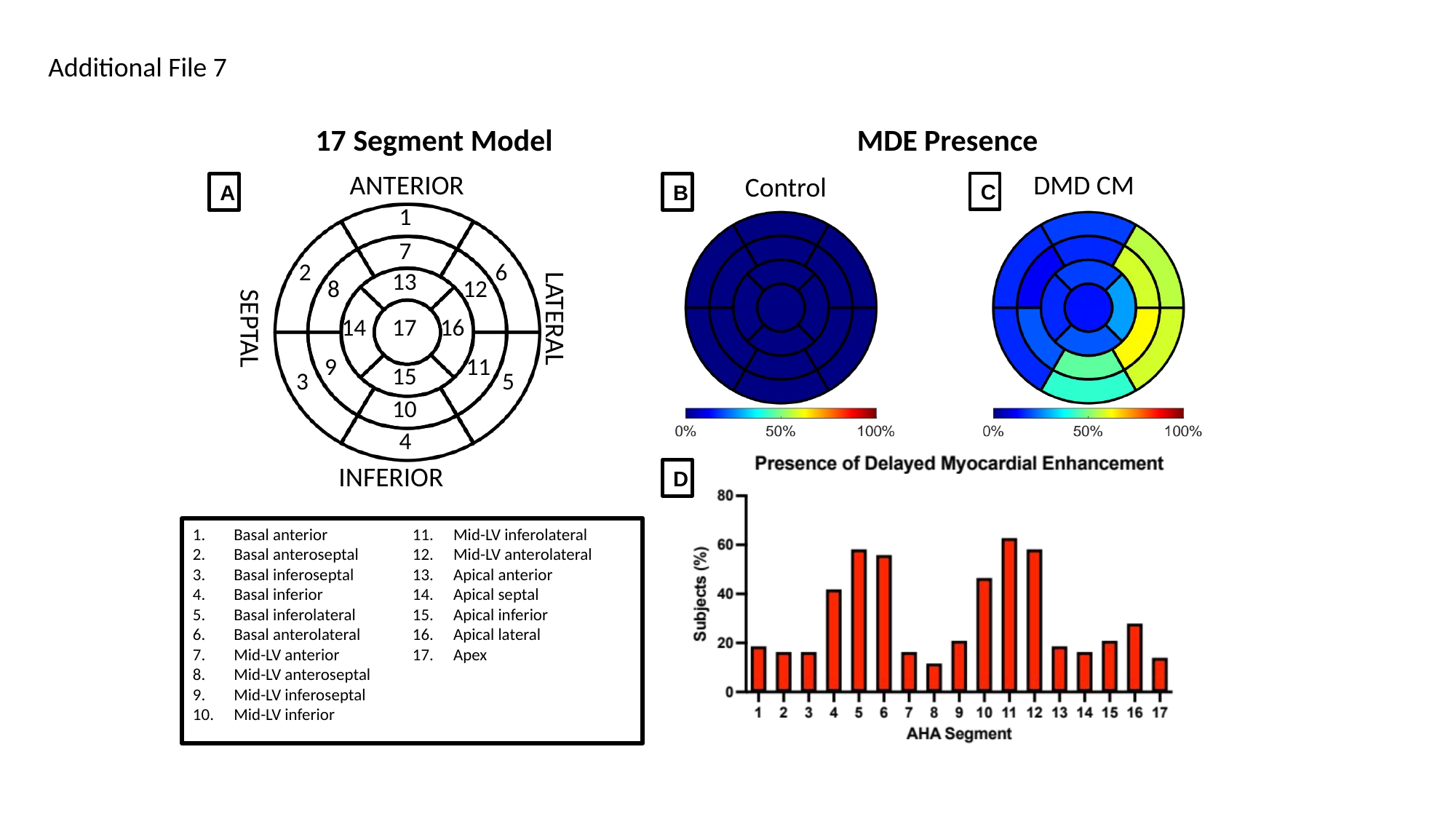

Additional File 7
17 Segment Model
MDE Presence
DMD CM
ANTERIOR
1
7
2
6
13
12
8
14
17
16
9
11
15
3
5
10
4
LATERAL
SEPTAL
INFERIOR
Control
C
A
B
D
Basal anterior
Basal anteroseptal
Basal inferoseptal
Basal inferior
Basal inferolateral
Basal anterolateral
Mid-LV anterior
Mid-LV anteroseptal
Mid-LV inferoseptal
Mid-LV inferior
Mid-LV inferolateral
Mid-LV anterolateral
Apical anterior
Apical septal
Apical inferior
Apical lateral
Apex
