# Supplementary material for: Localized strain characterization of cardiomyopathy in Duchenne muscular dystrophy using novel 4D kinematic analysis of cine cardiovascular magnetic resonance
Source: J Cardiovasc Magn Reson. 2023 Feb 16;25:14. doi: 10.1186/s12968-023-00922-3 (PMC9933368; doi:10.1186/s12968-023-00922-3)
Supplement: Supplementary file 8 — Additional file 8. Regional strain correlation (Pearson’s r) for peak strain and systolic strain rate. S- septal, FW- free-wall. [file 12968_2023_922_MOESM8_ESM.pptx]

## Slide 1
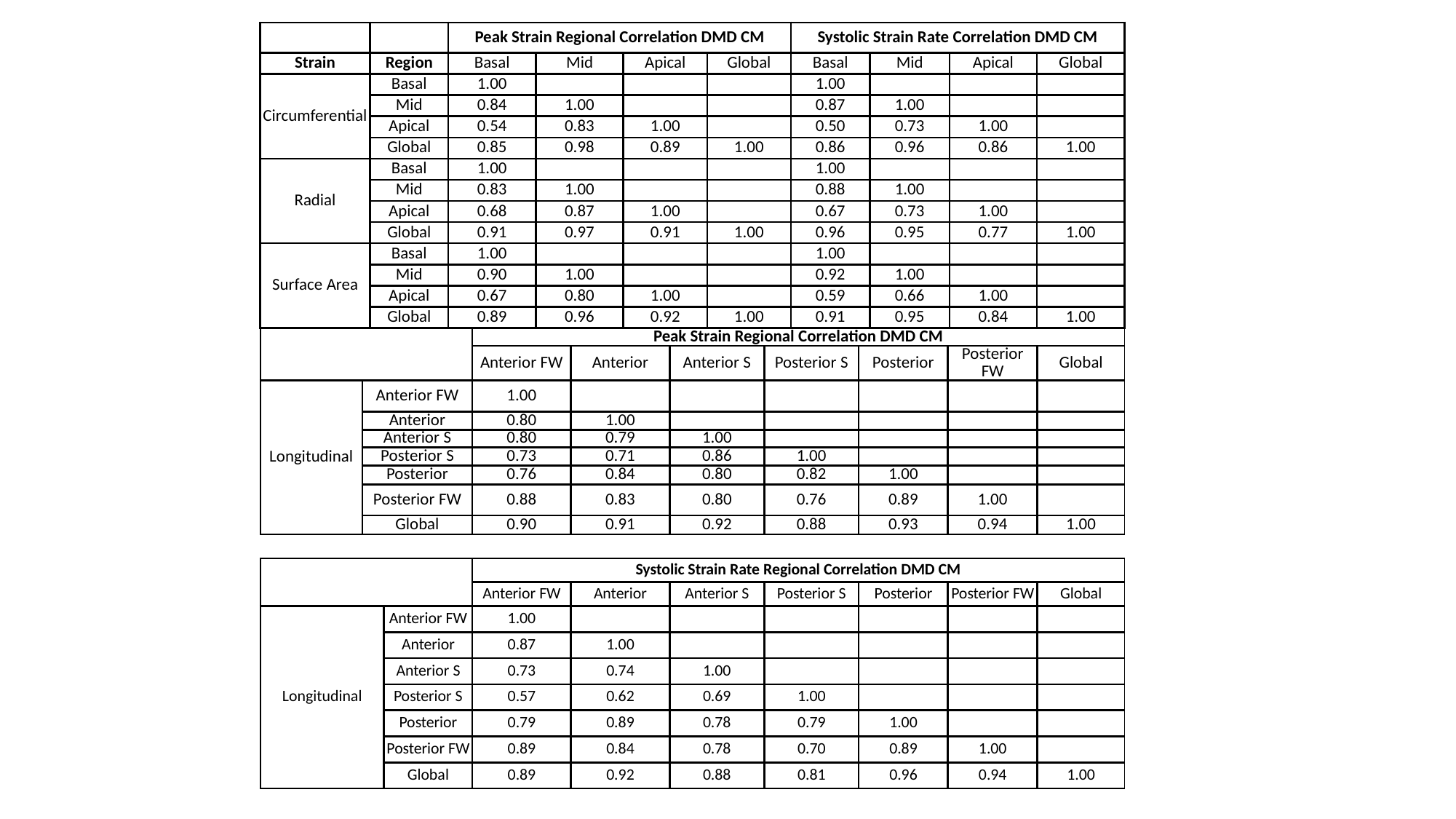

| | | Peak Strain Regional Correlation DMD CM | | | | Systolic Strain Rate Correlation DMD CM | | | |
| --- | --- | --- | --- | --- | --- | --- | --- | --- | --- |
| Strain | Region | Basal | Mid | Apical | Global | Basal | Mid | Apical | Global |
| Circumferential | Basal | 1.00 | | | | 1.00 | | | |
| | Mid | 0.84 | 1.00 | | | 0.87 | 1.00 | | |
| | Apical | 0.54 | 0.83 | 1.00 | | 0.50 | 0.73 | 1.00 | |
| | Global | 0.85 | 0.98 | 0.89 | 1.00 | 0.86 | 0.96 | 0.86 | 1.00 |
| Radial | Basal | 1.00 | | | | 1.00 | | | |
| | Mid | 0.83 | 1.00 | | | 0.88 | 1.00 | | |
| | Apical | 0.68 | 0.87 | 1.00 | | 0.67 | 0.73 | 1.00 | |
| | Global | 0.91 | 0.97 | 0.91 | 1.00 | 0.96 | 0.95 | 0.77 | 1.00 |
| Surface Area | Basal | 1.00 | | | | 1.00 | | | |
| | Mid | 0.90 | 1.00 | | | 0.92 | 1.00 | | |
| | Apical | 0.67 | 0.80 | 1.00 | | 0.59 | 0.66 | 1.00 | |
| | Global | 0.89 | 0.96 | 0.92 | 1.00 | 0.91 | 0.95 | 0.84 | 1.00 |
| | | Peak Strain Regional Correlation DMD CM | | | | | | |
| --- | --- | --- | --- | --- | --- | --- | --- | --- |
| | | Anterior FW | Anterior | Anterior S | Posterior S | Posterior | Posterior FW | Global |
| Longitudinal | Anterior FW | 1.00 | | | | | | |
| | Anterior | 0.80 | 1.00 | | | | | |
| | Anterior S | 0.80 | 0.79 | 1.00 | | | | |
| | Posterior S | 0.73 | 0.71 | 0.86 | 1.00 | | | |
| | Posterior | 0.76 | 0.84 | 0.80 | 0.82 | 1.00 | | |
| | Posterior FW | 0.88 | 0.83 | 0.80 | 0.76 | 0.89 | 1.00 | |
| | Global | 0.90 | 0.91 | 0.92 | 0.88 | 0.93 | 0.94 | 1.00 |
| | | Systolic Strain Rate Regional Correlation DMD CM | | | | | | |
| --- | --- | --- | --- | --- | --- | --- | --- | --- |
| | | Anterior FW | Anterior | Anterior S | Posterior S | Posterior | Posterior FW | Global |
| Longitudinal | Anterior FW | 1.00 | | | | | | |
| | Anterior | 0.87 | 1.00 | | | | | |
| | Anterior S | 0.73 | 0.74 | 1.00 | | | | |
| | Posterior S | 0.57 | 0.62 | 0.69 | 1.00 | | | |
| | Posterior | 0.79 | 0.89 | 0.78 | 0.79 | 1.00 | | |
| | Posterior FW | 0.89 | 0.84 | 0.78 | 0.70 | 0.89 | 1.00 | |
| | Global | 0.89 | 0.92 | 0.88 | 0.81 | 0.96 | 0.94 | 1.00 |
