# Supplementary material for: Localized strain characterization of cardiomyopathy in Duchenne muscular dystrophy using novel 4D kinematic analysis of cine cardiovascular magnetic resonance
Source: J Cardiovasc Magn Reson. 2023 Feb 16;25:14. doi: 10.1186/s12968-023-00922-3 (PMC9933368; doi:10.1186/s12968-023-00922-3)
Supplement: Supplementary file 9 — Additional file 9. Peak and systolic strain rate regional correlation (Pearson’s r). significant AFW- Anterior Free Wall, A- Anterior, AS- Anterior Septal, PS- Posterior Septal, P- Posterior, PFW- Posterior Free Wall, P-Peak, SR-Strain Rate. [file 12968_2023_922_MOESM9_ESM.pptx]

## Slide 1
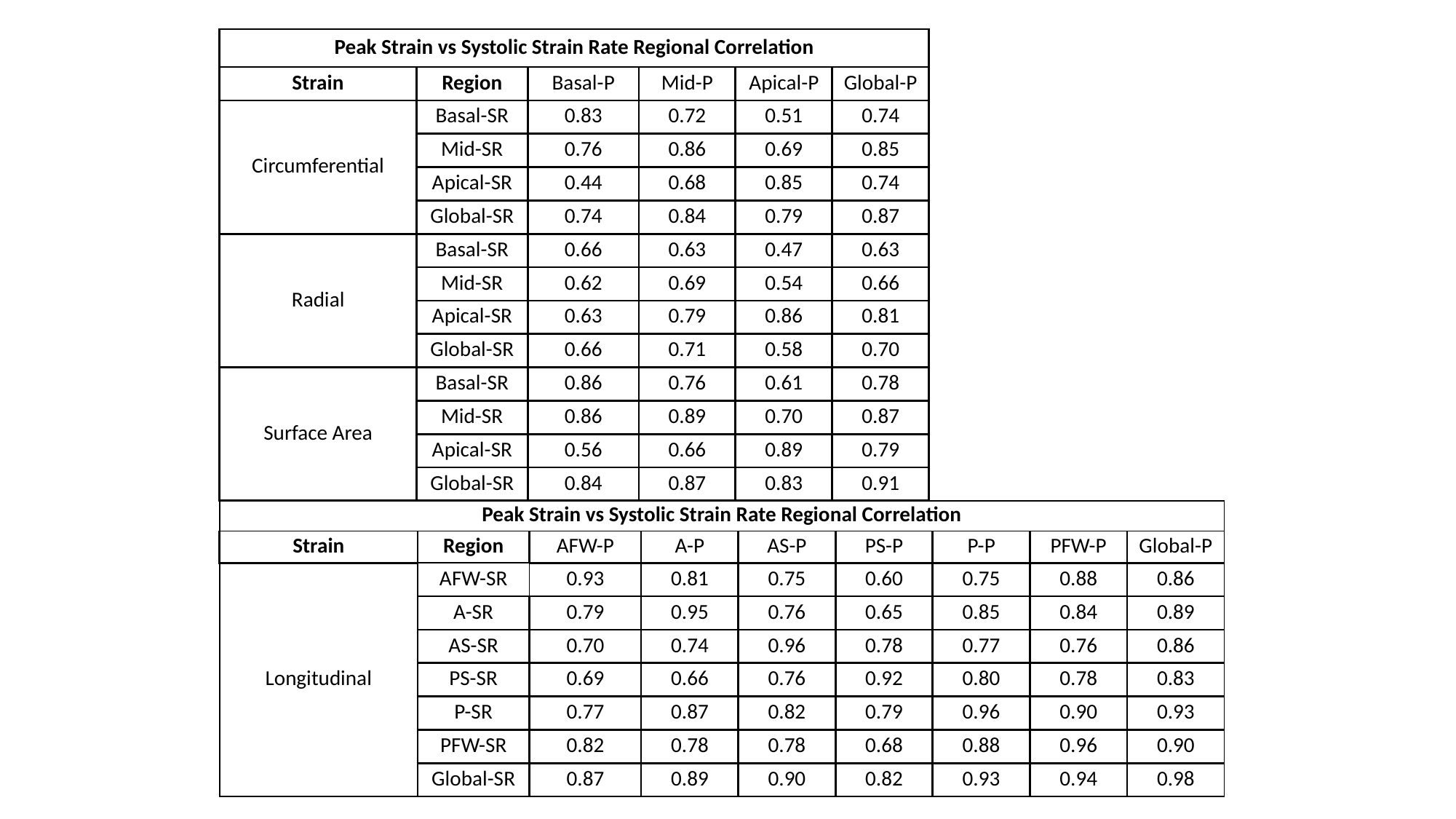

| Peak Strain vs Systolic Strain Rate Regional Correlation | | | | | |
| --- | --- | --- | --- | --- | --- |
| Strain | Region | Basal-P | Mid-P | Apical-P | Global-P |
| Circumferential | Basal-SR | 0.83 | 0.72 | 0.51 | 0.74 |
| | Mid-SR | 0.76 | 0.86 | 0.69 | 0.85 |
| | Apical-SR | 0.44 | 0.68 | 0.85 | 0.74 |
| | Global-SR | 0.74 | 0.84 | 0.79 | 0.87 |
| Radial | Basal-SR | 0.66 | 0.63 | 0.47 | 0.63 |
| | Mid-SR | 0.62 | 0.69 | 0.54 | 0.66 |
| | Apical-SR | 0.63 | 0.79 | 0.86 | 0.81 |
| | Global-SR | 0.66 | 0.71 | 0.58 | 0.70 |
| Surface Area | Basal-SR | 0.86 | 0.76 | 0.61 | 0.78 |
| | Mid-SR | 0.86 | 0.89 | 0.70 | 0.87 |
| | Apical-SR | 0.56 | 0.66 | 0.89 | 0.79 |
| | Global-SR | 0.84 | 0.87 | 0.83 | 0.91 |
| Peak Strain vs Systolic Strain Rate Regional Correlation | | | | | | | | |
| --- | --- | --- | --- | --- | --- | --- | --- | --- |
| Strain | Region | AFW-P | A-P | AS-P | PS-P | P-P | PFW-P | Global-P |
| Longitudinal | AFW-SR | 0.93 | 0.81 | 0.75 | 0.60 | 0.75 | 0.88 | 0.86 |
| | A-SR | 0.79 | 0.95 | 0.76 | 0.65 | 0.85 | 0.84 | 0.89 |
| | AS-SR | 0.70 | 0.74 | 0.96 | 0.78 | 0.77 | 0.76 | 0.86 |
| | PS-SR | 0.69 | 0.66 | 0.76 | 0.92 | 0.80 | 0.78 | 0.83 |
| | P-SR | 0.77 | 0.87 | 0.82 | 0.79 | 0.96 | 0.90 | 0.93 |
| | PFW-SR | 0.82 | 0.78 | 0.78 | 0.68 | 0.88 | 0.96 | 0.90 |
| | Global-SR | 0.87 | 0.89 | 0.90 | 0.82 | 0.93 | 0.94 | 0.98 |
